# State of Charge and State of Health Assessment of Viologens in Aqueous‐Organic Redox‐Flow Electrolytes Using In Situ IR Spectroscopy and Multivariate Curve Resolution

**DOI:** 10.1002/advs.202200535

**Published:** 2022-04-28

**Authors:** Oliver Nolte, Robert Geitner, Ivan A. Volodin, Philip Rohland, Martin D. Hager, Ulrich S. Schubert

**Affiliations:** ^1^ Laboratory of Organic and Macromolecular Chemistry (IOMC) Friedrich Schiller University Jena Humboldtstr. 10 07743 Jena Germany; ^2^ Jena Center for Soft Matter (JCSM) Friedrich Schiller University Jena Philosophenweg 7 07743 Jena Germany; ^3^ Center for Energy and Environmental Chemistry Jena (CEEC Jena) Friedrich Schiller University Jena Philosophenweg 7a 07743 Jena Germany; ^4^ Institute of Chemistry and Bioengineering Technical University Ilmenau Weimarer Str. 32 98693 Ilmenau Germany

**Keywords:** electrolyte state assessment, IR spectroscopy, multivariate curve resolution, redox flow battery, viologen

## Abstract

Aqueous‐organic redox flow batteries (RFBs) have gained considerable interest in recent years, given their potential for an economically viable energy storage at large scale. This, however, strongly depends on both the robustness of the underlying electrolyte chemistry against molecular decomposition reactions as well as the device's operation. With regard to this, the presented study focuses on the use of in situ IR spectroscopy in combination with a multivariate curve resolution approach to gain insight into both the molecular structures of the active materials present within the electrolyte as well as crucial electrolyte state parameters, represented by the electrolyte's state of charge (SOC) and state of health (SOH). To demonstrate the general applicability of the approach, methyl viologen (MV) and *bis*(3‐trimethylammonium)propyl viologen (BTMAPV) are chosen, as viologens are frequently used as negolytes in aqueous‐organic RFBs. The study's findings highlight the impact of in situ spectroscopy and spectral deconvolution tools on the precision of the obtainable SOC and SOH values. Furthermore, the study indicates the occurrence of multiple viologen dimers, which possibly influence the electrolyte lifetime and charging characteristics.

## Graphical abstract

The accurate assessment of battery state variables in organic redox flow batteries is crucial for a long service life. Using in situ IR spectroscopy and a multivariate curve resolution‐alternating least squares (MCR‐ALS) algorithm, different viologen scaffolds are analyzed, enabling the extraction of the state of charge (SOC), state of health (SOH) parameters and the identification of possible solution structures.

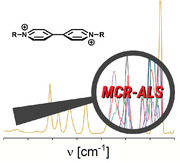

## 1 Introduction

Global energy‐related carbon dioxide emissions are currently at a staggering 35 Gt of CO_2_ (three quarters of the global total), contributing significantly to the climate crisis.^[^ ^1^ ^]^ Renewables on the other hand, are considered to be a key technology for a transition toward the decarbonization of electricity grids around the world. Within the last decade, in particular wind and solar installations became economically more viable, with a further drop in installation costs projected over the coming years.^[^ ^2^ ^]^ However, the intrinsic intermittency of the energy output of these renewables represents a major challenge to energy grids and requires a simultaneous installation of energy storage capabilities on a large scale in order to handle shifting demands and supplies.^[^ ^3^ ^]^ Among suitable storage concepts, redox flow batteries (RFBs) represent a promising technology, allowing for a theoretically independent scalability of energy and power output.^[^ ^4^ ^]^ Up until now, various RFB chemistries have been presented in the scientific literature, of which vanadium‐based batteries represent the most advanced systems in terms of commercialization.^[^ ^5^ ^]^ Nevertheless, due to various reasons, including the strong dependency on commodity prices for these systems, their overall market share is projected to remain low.^[^ ^6^ ^]^ This drawback may be overcome by the use of inexpensive organic redox‐active materials, which, however, suffer from stability issues, influencing the lifetime of these materials.^[^ ^7^ ^]^ Therefore, as pointed out in recent studies,^[^ ^8^ ^]^ it is crucial to have exact knowledge of the electrolyte state variables state of charge (SOC) and state of health (SOH) at any given point in time to enable a safe and efficient operation over several years. In order to determine these electrolyte state variables, online sensors for the measurement of the electrolyte redox potential or UV/Vis spectroscopic measurement cells are commonly used, although other approaches have been presented as well.^[^ ^8b^ ^]^

**Scheme 1:**
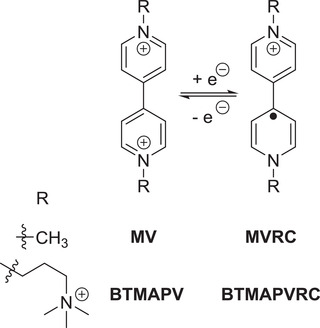
Schematic representation of the electrochemistry, structure and naming of (charged) viologen compounds used within this study: MV = methyl viologen; MVRC = methyl viologen radical cation; BTMAPV = bis(3‐trimethylammonium)propyl viologen; BTMAPVRC ≅ radical cation.

In a previous publication from our group, we introduced the concept of using an online IR setup for the in situ determination of individual species concentrations within RFB electrolytes, out of which both the SOC and SOH parameters could be obtained, using the well‐known *N*,*N*,*N*‐2,2,6,6‐heptamethylpiperidinyl oxy‐4‐ammonium chloride (TEMPTMA) molecule as a model substance.^[^ ^9^ ^]^ The concept was based on the use of FT‐IR spectroscopy in combination with an attenuated total reflection (ATR) cell, using a compositionally symmetric open circuit voltage (OCV) cell, as used in the publications of Ressel and Stolze, as reference.^[^ ^10^ ^]^ The presented analysis approach based on the Lambert–Beer law enabled the easy and fast measurement of species concentrations, even at the comparably high concentrations (>1 m) typically used in RFB electrolytes. Within the present publication, we transfer the usage of in situ IR spectroscopy to another, albeit more challenging, redox‐active organic structure useful for RFB electrolytes. These are represented by viologens, which are typically used as negolytes in aqueous‐organic RFBs.^[^ ^11^ ^]^ Therefore, we selected two promising candidates that have been frequently used within the scientific literature: The “standard” methyl viologen dichloride (MV)^[^ ^12^ ^]^ and *bis*(3‐trimethylammonium)propyl viologen tetrachloride (BTMAPV), which has been described in literature as a very stable active material (see **Scheme** **1**).^[^ ^13^ ^]^ These were investigated using an unbalanced compositionally symmetric test cell setup^[^ ^14^ ^]^ with a constant monitoring of the capacity‐limiting electrolyte by online IR spectroscopic measurements. In order to deduce individual species concentrations from the measurement and, hence, be able to predict the SOC and SOH, a new analysis approach based on multivariate curve resolution in combination with a non‐negative least squares fit was developed. This approach furthermore enabled insights into the molecular structures of the species present within the electrolytes. Furthermore, a UV/Vis analysis was conducted to estimate the binding constants for the radical‐cation dimers of both viologens.

## 2 Results and Discussion

Viologens can undergo two one‐electron redox processes, of which usually only the first is considered to be fully reversible (see Scheme 1).^[^ ^14a^ ^]^ With a typical redox potential for the first reduction step of around ‐0.45 V versus NHE,^[^ ^11^, ^15^ ^]^ viologens are generally used on the anolyte side of aqueous RFBs, e.g., coupled with posolytes based on (2,2,6,6‐tetramethylpiperidin‐1‐yl)oxyl (TEMPO) or ferrocene.^[^ ^12^, ^13^ ^]^ However, the negative redox potential also results in a sensitivity toward molecular oxygen, requiring a handling under inert conditions.

During our experiments with MV, we found that the initially used symmetric OCV cell did not deliver reliable estimates for the redox potential of the capacity‐limiting electrolyte, as shown in Figure S1 (Supporting Information). The graph depicts a clear discrepancy between the recorded data and the fit according to the Nernst equation, with the strongest deviation at high SOCs. These observations are plausible for a coupled electrochemical reaction, in which the product does show another chemical equilibrium and its decreased activity is, thus, affecting the Nernst potential. It is known that viologens are able to dimerize upon reduction to the radical‐cationic form, due to *π*–*π*‐interactions (so‐called *π*‐stacking) between the aromatic rings.^[^ ^16^ ^]^ This may change the concentration of the radical‐cationic species present in the electrolyte and thus affect the redox potential of the electrolyte. Interestingly, this may as well be the case for other redox couples of interest, such as the V^IV^/V^V^ or hydroquinone/quinone couples, for which species interactions are known as well.^[^ ^10^, ^17^ ^]^

As discussed within our previous study, which established the use of in situ IR spectroscopy for the online monitoring of RFBs,^[^ ^9^ ^]^ any of the recorded IR spectra have been subjected to a pre‐processing scheme that was designed to minimize the influences of water vapor and temperature changes, correct for the wavelength‐dependent penetration depth in the ATR setup as well as provide a background correction for the solvent (and any supporting electrolyte). Detailed information on the preprocessing steps is available in the Supporting Information.

**Figure 1:**
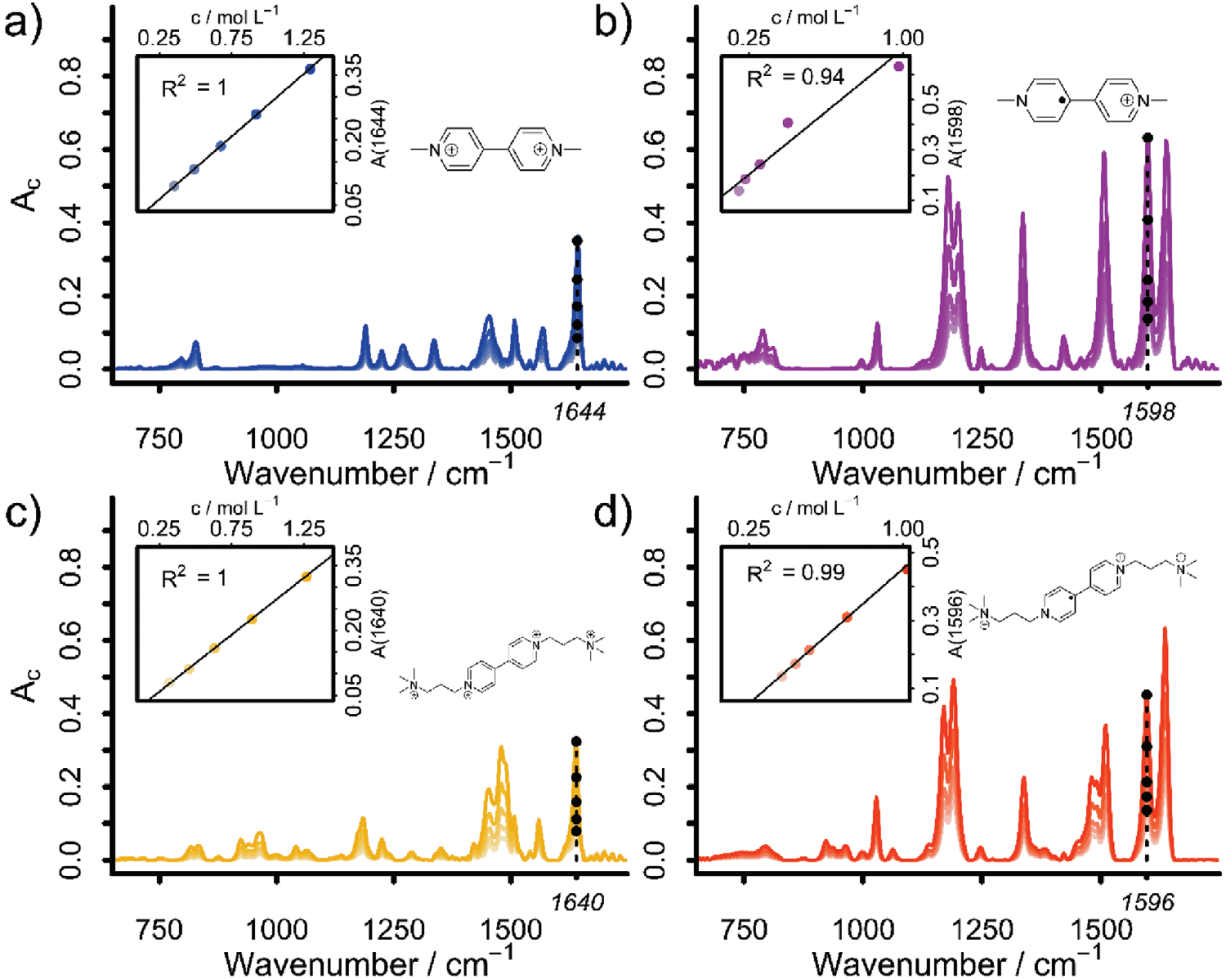
Baseline and ATR corrected IR spectra of MV (a, blue), MVRC (b, pink), BTMAPV (c, yellow) and BTMAPVRC (d, red) with varying concentrations. The inset shows the absorbances at 1644 (MV), 1598 (MVRC), 1640 (BTMAPV) and 1596 cm^−1^ (BTMAPVRC) plotted against concentration. Counterions have been omitted. The color intensity is indicative of the respective concentration, with more saturated colors implying higher concentrated solutions.

To test the feasibility of the previously established Lambert‐Beer approach for the analysis of viologen based RFBs, it is necessary to deduce the IR extinction coefficients for specific vibrations from a series of calibration measurements (dilution series using a (electrochemically reduced) stock solution of the respective compound in water, see Supporting Information). Representative IR spectra of the respective fully charged and discharged species can be seen in **Figure** **1**.

The recorded IR calibrations show a high linearity, as indicated by the coefficient of determination at each wavenumber visible in Figure S2 (Supporting Information). In accordance with the approach presented in our previous study,^[^ ^9^ ^]^ the refractive index of all solutions was also recorded, as it is crucial for the SOH determination (see Supporting Information).

**Figure 2:**
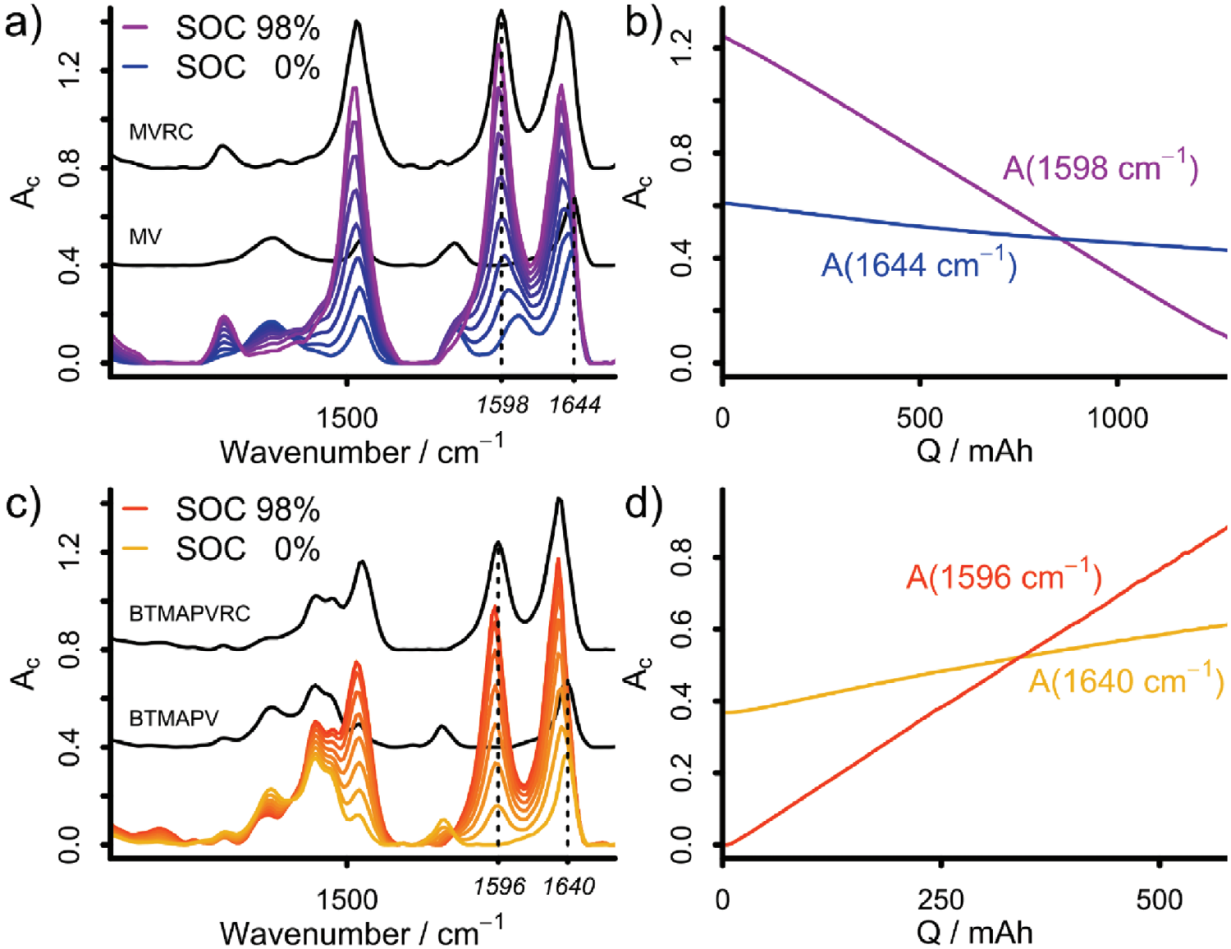
a,c) Selected baseline and ATR corrected IR spectra recorded in situ during the galvanostatic (dis)charging of an unbalanced compositionally symmetric a) MV/MVRC or BTMAPV/BTMAPVRC c) RFB. For reference, the IR spectra of an aqueous MV, MVRC, BTMAPV and BTMAPVRC solution are also shown, respectively. b,d) Selected IR absorbances recorded during galvanostatic discharging of b) a MV/MVRC‐based RFB and charging of d) a BTMAPV/BTMAPVRC‐based RFB.

However, as can be seen from **Figure** **2** in case of MV, using only the IR absorption at one specific wavenumber can lead to a nonlinearity in the absorption behavior. Furthermore, as can be anticipated from the literature survey and supported by the conducted OCV measurements,^[^ ^16^ ^]^ the viologen radical cations yielded by a one‐electron reduction of the viologen core may participate in a subsequent side reaction. This is characterized as an equilibrium that leads to the formation of a viologen radical cation dimer through *π*–*π* interactions. This coupled equilibrium will eventually decrease the concentration of the respective radical cation within the electrolyte mixture and, thus, cause an additional systematical error. Additionally, one cannot assume that only one single species is present in the fully charged electrolyte, as the electrolyte at this SOC will likely consist of both monomeric radical cations and their respective dimeric counterparts. This creates the challenge that any extinction coefficients derived for the respective radical cationic species may not be representing single species. Therefore, it can be concluded that the rather simple analysis approach based on the Lambert–Beer law is not transferrable to the analysis of IR spectra of more complex reactions.

To overcome this challenge, a new data analysis approach based on a multivariate curve resolution‐alternating least squares (MCR‐ALS) algorithm and subsequent nonnegative least squares (NNLS) fits was developed. The MCR‐ALS algorithm decomposes the input matrix *D* into a spectral matrix *S* and a concentration matrix *C* by minimizing the error matrix *E*.^[^ ^18^ ^]^

(1) $$D=C\cdot S^{T}+E$$

This is performed in an alternating fashion by first optimizing *S* and leaving *C* constant and vice versa until a specified stop criterion is reached. The only variable in the optimization procedure is the number of compounds *n* used to decompose the input matrix *D*. The idea behind this approach is to train the MCR‐ALS algorithm on a set of in situ IR spectra (*D*) recorded during charging and discharging of the viologen‐based electrolyte. This training step yields a spectral matrix (*S*), containing the IR spectra of the specified number of compounds *n*. In this study, we used 1000 in situ IR spectra as training set for each RFB system. To solve the MCR‐ALS equation during training, we further applied the constraint that the total electrolyte concentration in the monitored half‐cell is constant which is a reasonable assumption as the dataset was recorded under stable external conditions. Further constraints for the MCR‐ALS algorithm were non‐negativity for *S* and *C* which is equivalent to minimal absorbances and concentrations of zero. Subsequently, the extracted IR spectra were used in NNLS fits on a new set of in situ IR spectra which were not used during training to calculate the time‐dependent concentration of each spectral component. NNLS fits are commonly used in spectroscopy^[^ ^19^ ^]^ as they enable setting constraints on the fitting procedure (e.g., non‐negativity to prohibit negative concentrations, as they are not observed in chemistry). Thus, the result of the MCR‐ALS/NNLS‐based analysis approach is an IR spectrum for each component as well as its concentration at each point in time.

**Figure 3:**
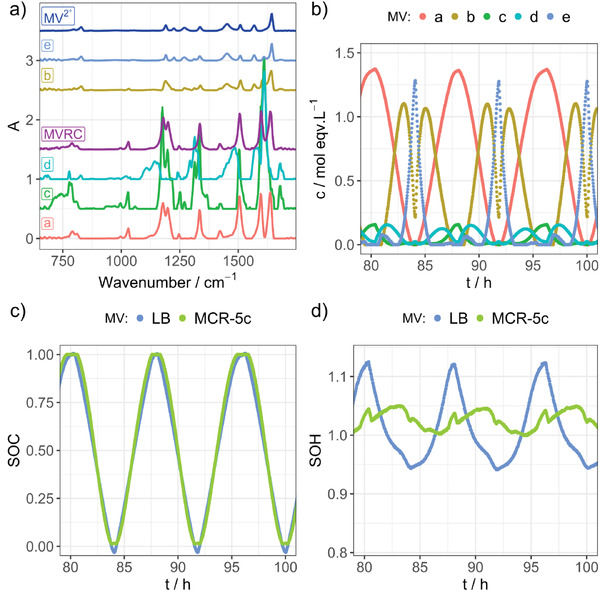
Results of the MCR‐ALS/NNLS algorithms using five components for an MV RFB. a) IR spectra of components a–e as well as the IR spectra of MV and MVRC as references (individual spectra offset used for readability). b) Concentration profiles for MCR components a–e. c,d) SOC and SOH values calculated using the concentrations of charged and uncharged species as extracted by the Lambert–Beer (LB) and MCR‐ALS/NNLS algorithms (MCR‐5c). Both SOC curves are very close to the expected linear trend, while the SOH curve of the MCR‐ALS/NNLS approach shows significantly less variation.

**Figure 4:**
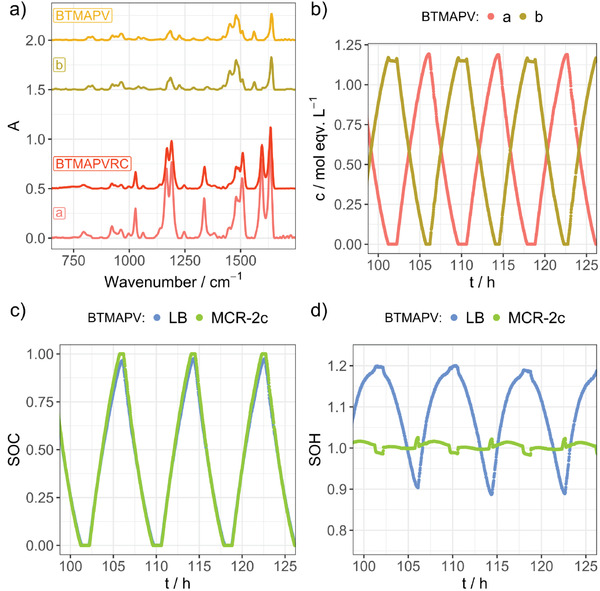
Results of the MCR‐ALS/NNLS algorithms using two components for a BTMAPV RFB. a) IR spectra of components a,b as well as the IR spectra of BTMAPV and BTMAPVRC as references (individual spectra offset used for readability). b) Concentration profiles for MCR components a,b. c,d) SOC and SOH values calculated using the concentrations of charged and uncharged species as extracted by the Lambert–Beer (LB) and MCR‐ALS/NNLS algorithms (MCR‐2c). Both SOC curves are very close to the expected linear trend, while the SOH curve of the MCR‐ALS/NNLS approach shows significantly less variation.

The results from the MCR‐ALS/NNLS approach and their comparison to the Lambert‐Beer‐based evaluation can be seen in **Figure** **3** (MV/MVRC, see Figures S3–S5, Supporting Information for extended representations with less overlap) and **Figure** **4** (BTMAPV/BTMAPVRC, see Figures S10 and S11, Supporting Information for extended representations with less overlap). In this study, the number of components, *n*, was varied in whole numbers ranging from two to six, to find the most suitable data interpretation (see Figures S6–S9, Supporting Information (MV/MVRC) as well as Figures S12–S15, Supporting Information (BTMAPV/BTMAPVRC)). In case of MV, five components were needed for a reasonable SOH representation. In contrast to this, the BTMAPV system yielded the best results when only two components were used within the MCR‐ALS/NNLS analysis. Panels a) in both Figures 3 and 4 depict the extracted IR spectra for each species in addition to the reference spectra of the fully charged and discharged electrolytes of the respective viologens. One can see that for the BTMAPV system, the extracted spectra are in close agreement with the reference spectra. While this is partially true for the MV system as well (see IR spectra of components a, b, and e in Figure 3a), the analysis also yields two IR spectra, which show signals not present in either reference spectrum.

Panels b in Figures 3 and 4 show an excerpt of the concentration profiles for each component during multiple charging and discharging cycles (for an extended time period see Figures S5 and S11, Supporting Information). For the MV/MVRC system, components a, b, and e feature maximum concentrations above 1 m while the maximum concentration of components c and d only reaches 0.2 m. For the BTMAPV RFB, the case is simpler as both compounds make up the total electrolyte concentration when the RFB is either fully charged (a) or fully discharged (b).

To derive time‐dependent SOC and SOH values from the extracted concentration profiles, each species needs to be assigned as either a charged or an uncharged species to consider its contribution to the electrochemical state of the battery. To realize this assignment, the IR spectra of each component were compared to the reference IR spectra of their respective fully charged and discharged electrolytes using the Pearsons correlation coefficient. When the correlation coefficient between the components’ IR spectrum and the IR spectrum of the uncharged reference component was larger than the correlation with the IR spectrum of the charged reference component, the component in question was assigned as an uncharged component and vice versa. For the MV/MVRC system, this led to the assignment of components a, c, and d as charged components while b and e were assigned as uncharged. For the BTMAPV/BTMAPVRC RFB, component a was assigned as charged while b was assigned uncharged.

With these assignments at hand, SOC and SOH values for the battery systems can be calculated. For the SOC, the total electron‐equivalent concentration of all charged species, ∑*c*_i,charged_, is divided by total electron‐equivalent concentration of all present electroactive species, ∑*c*_i_, while the SOH is defined as the present total electrolyte electron‐equivalent concentration in reference to the total electrolyte electron‐equivalent concentration at a reference point in time, ∑*c*_i,ref_,:

(2) $$\mathrm{SOC}=\frac{\sum c_{i,\mathrm{charged}}}{\sum c_{i}}$$

(3) $$\mathrm{SOH}=\frac{\sum c_{i}}{\sum c_{i,\mathrm{ref}}}$$

As the MCR‐ALS/NNLS analysis yields a concentration for each species and point in time, both SOC and SOH for the studied half‐cell can be deduced from the data. Panels c and d of Figure 3 depict the respective SOC and SOH values for the MV system, based on the best‐fitting MCR‐ALS/NNLS data, while Figure 4 shows these data for the BTMAPV system. In addition, the respective panels also display the SOC and SOH values derived from analysis approach based on the Lambert–Beer law (LB). Regarding the SOC, the MCR‐ALS/NNLS‐based approach only yields minimal improvements over the established LB approach. The reason for the improvement is the fact that the MCR‐ALS/NNLS approach relies on the use of a complete IR spectrum representing all wavenumber positions, while the LB approach only uses absorbances at selected wavenumber positions. This finding indicates that the chosen wavenumber positions in the LB approach are very good descriptors for the SOC of the RFB systems. This is expected as the handpicked IR absorbances at 1596 (MVRC) and 1598 cm^−1^ (BTMAPVRC) are only present in the viologen radical cation species while also featuring high IR extinction coefficients. These properties make the chosen wavenumber positions ideal for following the RFBs SOC (see Figure 2).

In contrast to the SOC determination, the SOH measurements profit significantly from the MCR‐ALS/NNLS approach when compared to the LB approach. The LB analysis leads to strongly varying SOH values: For the MV/MVRC system, the SOH varies from 94% to 112%, while a fluctuation from 90% to 120% for the BTMAPV/BTMAPVRC system can be observed when the expected SOH value is 100%. As can be seen in Figures 3d and 4d, the SOH varies systemically with the SOC of the RFB system. This behavior was also observed within our previous analysis of the TEMPTMA system, albeit to a much lesser extent.^[^ ^9^ ^]^ The MCR‐ALS/NNLS analysis reduces the variability range of the SOH values to 5%, which is a significant improvement over the ranges of 28 or 30% produced by the LB approach, respectively.

As for the SOC parameter, the reason for this improvement is most likely found in the utilization of all wavenumber positions for the concentration determination of each individual component, as the SOH analysis also improves when the MCR‐ALS/NNLS algorithm only uses two components like in the case of BTMAPV/BTMAPVRC. However, this effect is much larger for the SOH determination, as this parameter relies on concentrations deduced from two IR spectra, whereas the SOC can be determined from a single IR spectrum. Thus, an increase in accuracy benefits the precision of the SOH determination considerably more than it benefits the SOC determination when compared to the LB analysis.

**Figure 5:**
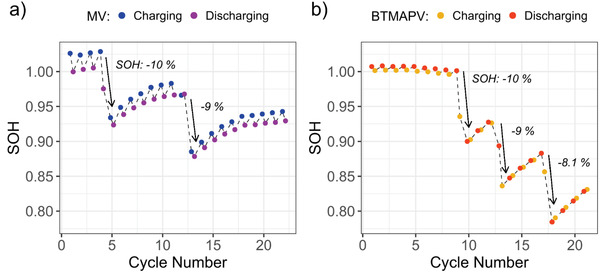
a) IR derived SOH for a MV RFB averaged overcharging (blue) and discharging half‐cycles (purple). b) IR derived SOH for a BTMAPV RFB averaged overcharging (yellow) and discharging half cycles (red). The SOH is artificially lowered by 10% in both cases multiple times. The SOH recovers due to crossover in the symmetrical RFBs.

**Figure** **5** depicts the SOH averaged over each individual charging and discharging half‐cycle. As can be seen, the SOH was artificially lowered multiple times by 10% by replacing part of the electrolyte solution with water. For both the MV/MVRC as well as the BTMAPV/BTMAPVRC system, the combination of in situ IR spectroscopy with a subsequent MCR‐ALS/NNLS data analysis approach is able to track changes in the SOH. The IR‐based analysis reveals a recovery of the SOH for both systems. In contrast to the IR‐based analysis, the SOH derived from the total battery capacity deduced via Coulomb counting does not show this SOH recovery (see Figure S19, Supporting Information).

The reason for this superficially contradicting result is the microscopic explanation for the SOH recovery. The IR‐measured SOH values are based on concentration measurements and, thus, a SOH recovery is equivalent to an electrolyte concentration increase after the concentration was artificially lowered. Simultaneously, the SOH derived by Coulomb counting is based on the total amount of the electroactive species within the studied half‐cell. As this parameter does not change further after part of the electrolyte solution was removed (species crossover neglected), the amount of electrolyte molecules also stays constant. Thus, the explanation for the concentration changes is not the diffusion of viologen molecules across the separator but rather the diffusion of water molecules between the two half‐cells of the battery, eventually leading to a volumetric imbalance that may in an industrial setup be addressed by an adjustment of the ionic strength of the electrolyte or addition of further redox‐active material.

Besides the presented advantages of the MCR‐ALS/NNLS analysis for the in situ state assessment of organic RFBs, the approach also enables a molecular view onto the processes at work during (dis)charging of highly concentrated and thus possibly industrial relevant viologen RFBs. For the BTMAPV/BTMAPVRC system, the MCR‐ALS/NNLS algorithm already provides satisfying results when two components are modeled.

The IR spectra of the two MCR components are in very good agreement with the reference spectra of both uncharged and charged BTMAPV electrolytes (Figure 4a). In addition, the concentration profiles of both components (Figure 4b) show an inverse behavior, revealing that during (dis)charging one of the components is directly transformed into the other.

Our interpretation of these findings is that the electrochemical charging process of the BTMAPV system is indeed just based on two main components: the monomeric BTMAPV and its respective radical cation dimer.

This interpretation is supported by the extracted IR spectra. The bands at 1640 (*ν*_ring_), 1560 (*ν*_ring_), 1508 (*δ*_CH_), 1480 (*δ*_CH3_), 1452 (*δ*_CH_), 1226 (*ν*_N‐CH2_) and 964 cm^−1^ (*ν*_C–C_) can be assigned for BTMAPV while the bands at 1634 (*ν*_ring_), 1596 (*ν*_ring_), 1510 (*ν*_ring_), 1510 (*δ*_CH_), 1480 (*δ*_CH3_), 1190 (*ν*_N‐CH2_) and 922 cm^−1^ (*ν*_C–C_) can be assigned for BTMAPVRC, which supports the suggested molecular structures.^[^ ^20^ ^]^ In addition, the number of absorption bands observable by IR spectroscopy does not significantly increase when BTMAPV is reduced to BTMAPVRC, although the symmetry of the molecule is lowered from D_2h_ to C_2v_, which should lead to an increase in the number of IR‐active vibrations. An explanation for this finding is the formation of a BTMPVRC dimer, which features the same symmetry as a BTMAPV monomer and thus both species would show a comparable number of IR active vibrations.^[^ ^20a^ ^]^

**Scheme 2:**
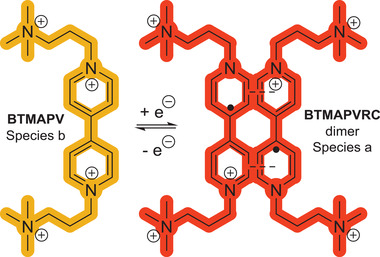
Schematic representation of the molecular structures found during (dis)charging of a BTMAPV/BTMAPVRC RFB based on MCR‐ALS/NNLS analyzed in situ IR spectra.

In order to verify the hypothesis of a dimer formation of the radical cations, we conducted UV/Vis experiments in analogy to the experiments conducted by the group of Winter, utilizing a chemical reduction with dithionite ions in order to generate the reduced radical‐cationic form (see Supporting Information).^[^ ^21^ ^]^ From the spectra (see Figure S26, Supporting Information), it is clearly visible that a radical cation dimer formation is taking place for BTMAPVRC, as evidenced by the evolution of a maximum at around 860 nm. The association constant *K*_a_ was determined to be 6.8  ×  10^1^ L mol^−1^, suggesting an equilibrium that is shifted toward the dimeric species. For a fully charged electrolyte at an effective total concentration of 1 m, this suggests a concentration of the monomeric species of around 3.7 × 10^−3^ m. The concentration of the monomer may thus be too low to be detectable by the presented IR‐spectroscopic approach, explaining the absence of a distinct component. **Scheme** **2** summarizes the results of the MCR approach for the BTMAPV electrolyte.

In contrast to the BTMAPV/BTMAPVRC system, the MV/MVRC system shows a much more complex behavior. For this system, it is not sufficient to model just two or three components (see Figures S6 and S7, Supporting Information), as best results are achieved with five components.

As already mentioned, MVRC forms dimeric species via *π*–*π* stacking interactions.^[^ ^16^ ^]^ Similar to the UV/Vis experiments conducted for BTMAPV, these studies were repeated for MV as well in order to deduce an association constant for the MV dichloride. As expected, the data (see Figure S27, Supporting Information) shows a *π*‐dimer formation even at low concentrations. The extracted association constant has a value of 5.48  ×  10^2^ L mol^−1^. This value is approximately an order of magnitude higher than that obtained for the measurement of the BTMAPV dimer and hints to an equilibrium that is strongly shifted toward the dimeric species. It is furthermore in the range of previously reported values,^[^ ^16^, ^21^, ^22^ ^]^ although differences may be attributed to the differing counter ions.^[^ ^16c^ ^]^ Based on the MCR‐ALS/NNLS‐extracted IR spectra and concentration profiles, components a and b were assigned to be the monomeric MV dication and the MV‐RC dimer, respectively, as supported by the conducted UV/Vis measurements. Both components have the highest concentration throughout the charging process and their IR spectra are very similar to the reference IR spectra recorded at full charge and discharge (e.g., 1640 (*ν*_ring_), 1570 (*ν*_ring_), and 1454 (*δ*_CH_) cm^−1^).^[^ ^20^, ^23^ ^]^ The symmetry argument also holds for MV. The number of IR observable bands does not increase, although the symmetry of the molecule is reduced when the radical cation forms. This again can be explained by the formation of the MVRC dimer.

While the aforementioned molecular structures assigned to components a and b are certainly present within the electrolyte, room for speculation about the molecular structures of components c, d, and e is given.

The gathered data for component c can be interpreted as belonging to a MVRC trimer formed via *π*–*π* stacking interactions, as it is only present at high SOC values. Its extracted IR bands at 1680, 1610, 1506, 1334, 1310, 1198, 1178, and 778 cm^−1^ are assigned as *ν*_ring_, *ν*_ring_, *δ*_CH_, *δ*_CH3_, *δ*_CH3_, *δ*_CH_+*ν*_ring_, and *γ*_CH_+Γ_ring_, based on the analysis of Poizat et al.,^[^ ^20a^ ^]^ Winter et al. also hinted at the existence of higher‐order aggregates for specific viologens.^[^ ^21^ ^]^

**Scheme 3:**
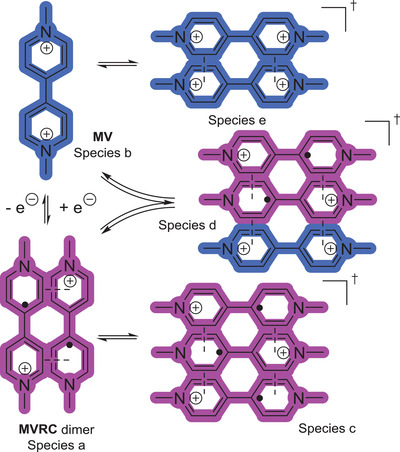
Suggested monomeric, dimeric, and trimeric molecular structures found during (dis)charging of a MV/MVRC RFB based on MCR‐ALS/NNLS analyzed in situ IR spectra.

The concentration of species e reaches its maximum when the SOC is close to 0%, leading us to speculate about an involvement of a dimeric form of the viologen dication. The associated IR spectrum is also very similar to the spectrum of the monomeric species b. This is to be expected as the interaction between the positively charged molecules should be lower than the *π*–*π*‐stacking of the MVRC. However, a MV dimer was not yet observed in other studies featuring lower concentrations,^[^ ^16b–d^ ^]^ but based on the MCR data, may be present at high concentrations in aqueous solutions. Finally, the concentration of species d peaks at SOC values of approximately 69%. Our hypothesis for the structure of this species is a mixed dimer formed by both MV and its radical cation MVRC, or even a trimer consisting of two MVRC molecules and one MV molecule. This hypothesis is backed by the extracted IR spectrum, which shows features of both the charged (1610 and 1314 cm^−1^) as well as uncharged (1576, 1486, and 826 cm^−1^) part of the MV/MVRC redox pair. The findings of the presented MCR approach for the investigated MV electrolyte are summarized in **Scheme** **3**. However, further spectroelectrochemical measurements may be needed to support the presence of these molecular structures. Additionally, we refrained from implementing a manual factorization according to the suggested stoichiometric ratios. Thus, the concentrations shown in Figures 3 and 4 should be regarded as electron‐equivalent concentrations.

The different behavior of the investigated redox pairs in the presented in situ IR study can be assigned to their different molecular structure. While BTMAPV bears four positive charges, partly located on flexible alkyl chains attached to the viologen core, MV only has two positive charges confined to its core. Due to the larger positive charge coupled with the flexibility of the alkyl chains, past reports suggested that BTMAPV does not form *π*‐dimers upon reduction.^[^ ^16c^ ^]^ However, both IR and UV/Vis investigations do show hints to doubt this hypothesis. As evidenced by the results of the MCR algorithm and supported by the UV/Vis analysis, BTMAPV does indeed form dimeric species upon reduction, suggesting that the majority of the BTMAPV radical‐cationic molecules are present as dimers. Nevertheless, the determined association constant is approx. one order of magnitude lower than that determined for the MV analog. Furthermore, BTMAPV does not seem to engage in the formation of association complexes of higher order, as has been hinted for MV.^[^ ^21^ ^]^ These observations may indeed be attributed to the increased intermolecular repulsive forces in BTMAPV. MV and its radical‐cation on the other hand, do experience less repulsive forces and are thus more likely to interact with each other in concentrated aqueous solutions, which can result in deviations of physicochemical parameters such as the solubility. Furthermore, as can be anticipated from the OCV data and has been shown before,^[^ ^16a^ ^]^ the formation of dimeric species can lead to a shift of the electrolytes’ redox potential, complicating electrochemical data analysis, which is part of ongoing investigations within our group. Besides this, further investigations connecting the presented results to the differences in observed capacity fade rates for electrolytes of both molecules, as pointed out by recent investigations,^[^ ^7^, ^13^, ^24^ ^]^ have to be conducted.

## 3 Conclusions

The presented study aimed at an in‐depth spectro‐electrochemical investigation of viologen‐based electrolyte systems for organic RFBs, using both methyl and BTMAP viologen due to their frequent use in the scientific literature. Regarding the complexity of the investigated electrolytes, a newly developed MCR‐ALS/NNLS analysis approach was used, which enables the spectral deconvolution of the recorded in situ IR spectra containing varying amounts of different species, yielding both spectra for each compound as well as their respective concentrations within the mixture. This data was then used to deduce the concentration‐based SOH of the electrolyte, which was artificially lowered over the experimental duration. Furthermore, the developed approach also improves the accuracy of IR‐based SOC measurements when compared to the rather simple analysis using only the Lambert–Beer law, which struggles with multicomponent systems. As for other spectroscopic approaches, the presented IR‐based approach is concentration‐based. As a consequence, a combination with Coulomb counting or volumetric sensors may improve the reliability and may enable the investigation of electrolyte transfer.

In addition to the aforementioned findings, the IR spectra processed by the new MCR‐ALS/NNLS analysis approach can be used to deduce structural information useful to identify molecular interactions within the viologen electrolytes. As was demonstrated, multiple molecular interactions seem relevant for the electrolyte system based on the MV/MVRC redox pair, while only dimers of the reduced radical‐cation are observed for the BTMAPV/BTMAPVRC system, as supported by the association constants for the respective radical cation dimers determined by UV/Vis spectroscopy. This molecular picture has thus far been underrepresented, but may be of importance for future work, in particular regarding the long‐term stability of viologen electrolytes. All in all, the presented approach leads to a more in‐depth understanding of viologen‐based RFB systems under realistic conditions, which enables a knowledge‐driven molecular optimization in the future.

While the analysis approach was demonstrated on viologen‐based RFB systems, it is not limited to this material class and can instead be applied to other systems, such as quinones or ferrocenes as well. Furthermore, it enables the study of decomposition reactions within RFB electrolytes in real‐time, as occurring species can be identified and quantified, which is part of future investigations.

## Conflict of Interest

The authors declare no conflict of interest.

## Supporting information

Supporting Information

## Data Availability

The data that support the findings of this study are available in the supplementary material of this article.
