# Supplementary material for: State of Charge and State of Health Assessment of Viologens in Aqueous‐Organic Redox‐Flow Electrolytes Using In Situ IR Spectroscopy and Multivariate Curve Resolution
Source: Adv Sci (Weinh). 2022 Apr 28;9(17):2200535. doi: 10.1002/advs.202200535 (PMC9189600; doi:10.1002/advs.202200535)
Supplement: Supplementary file 1 — Supporting Information [file ADVS-9-2200535-s001.pdf]

ADVANCED SCIENCE
Open Access

Supporting Information

for *Adv. Sci.*, DOI 10.1002/advs.202200535

State of Charge and State of Health Assessment of Viologens in Aqueous-Organic Redox-Flow Electrolytes Using In Situ IR Spectroscopy and Multivariate Curve Resolution

Oliver Nolte, Robert Geitner, Ivan A. Volodin, Philip Rohland, Martin D. Hager and Ulrich S. Schubert**

Supporting Information

State of Charge and State of Health Assessment of Viologens in Aqueous-organic Redox-Flow Electrolytes using in situ IR Spectroscopy and Multivariate Curve Resolution

Oliver Nolte, Robert Geitner Ivan A. Volodin, Philip Rohland, Martin D. Hager, Ulrich S. Schubert**

1. Experimental Details

Chloroacetic acid (Alfa Aesar, USA), 1-Bromo-3-chloropropan (TCI Germany GmbH, Germany), trimethylamine (3.2 M in methanol, TCI Germany GmbH, Germany), 4,4'-bipyridine (Alfa Aesar, USA), Dowex Marathon A2 (Sigma-Aldrich, Germany) were bought by the indicated supplier and used as received. NaCl 99.5% (laboratory grade) was bought from Fisher Chemical and used as received. The solvents were bought by standard suppliers and distilled prior to usage (diethyl ether, ethanol, acetone, dichloromethane) or used as received (dimethylformamide). 50 w/w % *N*,*N*,*N*-2,2,6,6-Heptamethylpiperidinyl oxy-4-ammonium chloride (TEMPTMA) in water was supplied by JenaBatteries GmbH, Germany. The synthesis of dimethyl viologen (MV) was performed based on the publication of Janoschka[1], while the synthesis of bis(3-trimethylammonio)propyl viologen tetrachloride (BTMAPV) was based on the strategies reported by Xu[2] and Liu[3].

Synthesis of 1,1'-dimethyl-4,4'-bipyridinium dichloride

4,4'-Bipyridine (200 g, 1.28 mol) and chloroacetic acid (266.2 g, 2.82 mmol) were dispersed in dimethylformamide (1 L) and mixed intensively with a KPG stirrer. After heating to 160 °C, the solids dissolved within approximately 1 h. Afterwards the reaction mixture was stirred for an additional 16 h at 160 °C. The resulting yellow-brown participate was filtered off after cooling to ambient temperature. The slightly yellow solid was dispersed in dimethylformamide (500 mL) and heated to 170 °C and stirred for an additional 1 h at 170 °C. Afterwards the suspension was filtered as hot as possible. The received solid was washed with acetone (3 × 200 mL) and subsequently with dichloromethane (3 × 200 mL). The desired product was received as off white powder (283 g, 85.9%) after drying for 48 h under reduced pressure (approx. 2-10 mbar) at 40 °C in a vacuum drying oven. ^{1}H NMR (D_2O, 300 MHz): δ [ppm] = 4.44 (6 H, s, N-CH_3), 8.47 (4 H, d, J = 6.59 Hz, $CH_{2(aromat.)}$), 8.99(4 H, d, J = 6.78 Hz, $CH_{2(aromat.)}N^+$).

Synthesis of 3-chloro-*N,N,N*-trimethylpropan-1-ammonium bromide

A mixture of 1-bromo-3-chloropropan (157 mL, 250 g, 1.6 mol) and 3.2 M trimethylamine solution in methanol (166 mL, 31 g, 0.53 mmol) was stirred for 24 h at ambient temperature. Afterwards diethyl ether (400 mL) was added to the reaction and stirred for 2 h. A white participate was formed and filtered off. The volume of the filtrate was reduced to approx. 200 mL under reduced pressure and diethyl ether (400 mL) was added. The white participate was filtered off, combined with the first fraction and washed with diethyl ether (3 x 200 mL) dried for 18 h under reduced pressure (approximately 2 to 10 mbar) at 40 °C in a Binder vacuum drying oven. The product was received as white crystalline powder (115 g, 99.9%). ^{1}H NMR (D_2O, 300 MHz): δ [ppm] = 2.24 (2 H, m, CH_2Cl-CH_2-CH_2NMe_3), 3.09 (9 H, s, CH_2Cl-CH_2-$CH_2N\mathit{Me}_3$), 3.47 (2 H, m, CH_2Cl-CH_2-CH_2NMe_3), 3.65 (2 H, t, J = 6.05 Hz, CH_2Cl-CH_2-CH_2NMe_3)

Synthesis of 1,1'-bis(3-(trimethylammonio)propyl)-4,4'-bipyridinium tetra chloride

4,4'-Bipyridine (30 g, 192 mmol) and 3-chloro-*N,N,N*-trimethylpropan-1-ammonium bromide (91.5 g, 423 mmol) were suspended in dimethylformamide (250 mL) and heated to 125 °C. Upon heating the solids completely dissolved and a yellow/brownish solution was formed. After stirring at 125 °C for 18 h the brownish participate was filtered off, washed with acetone (3 × 100 mL) and subsequently with dichloromethane (3 × 100 mL) before it was dried under reduced pressure. The solid was heated under reflux in ethanol (300 mL) and subsequently filtrated as a hot solution. The product was purified via an ion exchange with Dowex Marathon A2 (50 g) in deionized water (300 mL). Subsequently, the solvent was removed under reduced pressure. The desired product was received as white powder (36.1 g, 37.5%) after drying for 48 h under reduced pressure (approximately 2 to 10 mbar) at 40 °C in a Binder vacuum drying oven. ^{1}H NMR (D_2O, 300 MHz): δ [ppm] = 2.64 (4 H, m, CH_2BiPy-CH_2-CH_2NMe_3), 3.15 (18 H, s, CH_2BiPy -CH_2-$CH_2N\mathit{Me}_3$), 3.55 (4 H, m, CH_2BiPy-CH_2-CH_2NMe_3), 3.80 (4 H, t, J = 7.77 Hz, CH_2Cl-CH_2-CH_2NMe_3), 8.59 (4 H, d, J = 6.85 Hz, $CH_{2(aromat.)}$), 9.16 (4 H, d, J = 6.85 Hz, $CH_{2(aromat.)}N^+$); ^{13}C NMR (D_2O, 75 MHz): δ [ppm] = 24.62 (CH_2BiPy-CH_2-CH_2NMe_3), 53.21 (CH_2BiPy-CH_2-$CH_2N\mathit{Me}_3$), 58.26 (CH_2BiPy-CH_2-CH_2NMe_3), 62.40 (CH_2BiPy-CH_2-CH_2NMe_3), 127.50 ($CH_{2(aromat.)}$), 145.71 ($CH_{2(aromat.)}N^+$), 150.57 ($C_{(aromat.)}$).

2. Instruments

In situ infrared (IR) spectra were recorded using a liquid nitrogen-cooled ReactIR 701L (Mettler Toledo, Germany), equipped with a Micro Flow Cell DS DiComp (Mettler Toledo, Germany) between 650 and 4000 cm^{-1} at a resolution of 4 cm^{-1}. A background spectrum of deionized water was collected with 128 single scans prior to each experiment. Each individual *in situ* IR spectrum consists of 138 single scans, which corresponds to a measurement time of 90 s per spectrum. The same number of scans was used for the concentration calibration measurements of methyl viologen (MV) and 1,1'-bis(3-(trimethylammonium)propyl)-4,4'-bipyridinium tetra chloride (BTMAPV) and their respective radical cations (MVRC and BTMAPVRC).

For reference purposes, an ATR-IR spectra of a MV and BTMAPV crystals were recorded between 550 and 4000 cm^{-1} with a resolution of 4 cm^{-1} using a ZnSe crystal on an iD7-ATR-IR Nicolet iS5 (Thermo Scientific, Massachusetts). The IR spectrum consists of 8 individual measurements where each measurement is made up of 32 single scans.

The refractive indices of all solutions were measured using a digital ORF 85BM refractometer by KERN & SOHN GmbH (Balingen, Germany) inside a glove box.

3. Data Preprocessing

The IR spectra were preprocessed using R (4.0.3)[4] and the preprocessing scheme previously published.[5] The figures were made using the R packages ggplot2[6], dplyr[7] and gridExtra[8]. Firstly, the IR spectra were corrected for the influence of water vapor following a modified procedure from Bruun *et al.*.[9] For the correction only the wavenumber region from 1350 and 1840 cm^{-1} was used and corrected, respectively.

Following the water vapor correction, the IR spectra were corrected for the varying penetration depth of the IR light during the Attenuated Total Reflection (ATR) measurement mode. IR light of lower wavenumbers penetrates deeper into the sample than light of higher wavenumbers. Thus, the absorbance at lower wavenumbers is overestimated in ATR-IR compared to transmission IR. The concentration-dependent refractive index of all compounds in aqueous solution can be seen in **Table S3** to **Table S6** and **Figure S20** to **Figure S23**.

Subsequently, the IR spectra were cropped to the region of interest between 650 and 1750 cm^{-1}. Afterwards, the IR spectra were baseline-corrected using a SNIP algorithm[10,11] (iterations = 18, order = 2).

By using the Lambert-Beer-law, the wavenumber-dependent penetration depth *d* and the absorbances from the concentration calibration measurements *A*, it is possible to calculate the extinction coefficients of MV, MVRC, BTMAPV and BTMAPVRC in aqueous solution according to

$$\varepsilon = \frac{A}{c \cdot d} \qquad (1)$$

where *ε* is the extinction coefficient, *A* the concentration-dependent IR absorbance, *c* the concentration and *d* the penetration depth. The most important IR bands of all components as well as their extinction coefficients are summarized in **Table S1** and **Table S2**, respectively.

Table S1. Extinction coefficients of MV and its radical cation in the IR region. The extinction coefficients used for the concentration determination are highlighted as bold text.

MV bis-cation		MV radical cation	
ν [cm^{-1}]	ε [L mol^{-1} cm^{-1}]	ν [cm^{-1}]	ε [L mol^{-1} cm^{-1}]
**1644**	**2749**	1636	12652

1568	801	**1598**	**12398**
1508	1238	1506	10850
1454	1137	1422	1675
1336	576	1336	7953
1270	476	1248	1060
1224	347	1198	9198
1190	838	1178	9567
828	505	1030	2420
798	212	790	1899

Table S2. Extinction coefficients of BTMAPV and its radical cation in the IR region. The extinction coefficients used for the concentration determination are highlighted as bold text.

BTMAPV bis-cation		BTMAPV radical cation	
ν [cm^{-1}]	ε [L mol^{-1} cm^{-1}]	ν [cm^{-1}]	ε [L mol^{-1} cm^{-1}]
**1640**	**2481**	1634	5981
1560	1020	**1596**	**4023**
1508	1114	1510	3355
1480	2198	1480	2006
1452	1300	1338	1948
1350	206	1246	285
1288	195	1190	4665
1226	435	1170	3708
1184	840	1062	248
1042	257	1028	1568
964	497	922	447
924	376	796	336
834	262		

4. Data Analysis

An initial analysis was based on the previously described approach using a series of calibration measurements and Lambert-Beers law to extract the concentration of each species at every point in time from a series of IR spectra. This analysis worked well for the SOC determination but failed for an accurate SOH determination. Thus, a new analysis was developed.

The new approach is based on a Multivariante Curve Resolution – Alternating Least Square (MCR-ALS) algorithm as implanted by R package ALS.[12]

$$D = C \cdot S^T + E \qquad (2)$$

Where D is m x n data matrix (e.g. m IR spectra with n wavenumber positions recorded during RFB charging/discharging), C is a m x k concentration matrix, S^T a k x n spectra matrix containing the spectral fingerprint of k components and E is a m x n residual error matrix.

Firstly, 1000 IR spectra measured during galvanostatic charging and discharging of a symmetrical compositionally-unbalanced[13,14] MV/MVRC RFB with a total electrolyte concentration of 1.5 M were analyzed using MCR-ALS. Both the spectral as well as the concentration matrix were locked to non-negative values while the total concentration for each time point was locked to 1.5 M. The concentration matrix was initialized using the concentration values of MV and MVRC as extracted by the previous analysis based on Lambert-Beers law. The resulting spectra matrix S^T was subsequently used in a Non-Negative Least Square (NNLS) fit[15] to analyze a new set of *in situ* IR spectra. The result is a concentration matrix which contains the concentration of each species at each time point. Thus, the result of the combined use of MCR-ALS and NNLS algorithms is a set of IR spectra each representing a component in the RFB and the concentration of each component at every measurement time. The same procedure was applied for the *in situ* spectra recorded from a BTMAPV/BTMAPVRC RFB using 1.2 M as the total concentration during MCR-ALS analysis.

The MCR-ALS extracted spectra were compared to the reference spectra recorded during the calibration experiments using Pearson correlation coefficient.

When the spectrum of a MCR-ALS component matched the spectrum of MVRC it was assigned as charged species, while it was assigned as an uncharged species if it matched the spectrum of MV. A MCR-ALS spectrum matched MVRC when the corresponding correlation coefficient was higher

compared to the correlation coefficient between the component and MV. With this approach each MCR-ALS component was assigned either as a charged or uncharged species, respectively.

The SOC was calculated by comparing the concentration of all charged species to the total concentration of all species. The SOH was calculated by comparing the total concentration of all species to the total electrolyte concentration at a given reference point.

$$\mathrm{SOC} = \frac{\sum \mathrm{c_{i,charged}}}{\sum \mathrm{c_i}} \tag{3}$$

$$\mathrm{SOH} = \frac{\sum \mathrm{c_i}}{\sum \mathrm{c_{i,ref}}} \tag{4}$$

The number of components *k* in the MCR-ALS analysis can be freely chosen. The MCR-ALS/NNLS approach was tested using *k* = 2, 3, 4, 5 and 6 (see **Error! Reference source not found.**, **Error! Reference source not found.** as well as **Figure S3** - **Figure S9**). The quality of all MCR-ALS/NNLS fits was compared to the standard analysis offered by Lambert-Beers law. The quality of the respective MCR-ALS/NNLS fits was accessed based on a comparison to the recorded electrochemical data (voltage and current) for the SOC values. For the SOH analysis the aim was to achieve a low variability of the data as long as the SOH remained unchanged and to accurately represent the artificial drops in SOH induced throughout the experiment.

5. Refractive Index

Table S3. Concentration-dependent refractive index and IR absorbance of aqueous MV solutions.

c [mol L^{-1}]	n	A(1644 cm^{-1})
0.304	1.3470	0.081
0.356	1.3499	0.095
0.419	1.3555	0.112
0.492	1.3601	0.133
0.575	1.3638	0.158
0.676	1.3710	0.186
0.797	1.3766	0.220
0.921	1.3858	0.260
1.099	1.3978	0.307
1.293	1.4111	0.364
1.507	1.4224	0.431

Table S4. Concentration-dependent refractive index and IR absorbance of aqueous MVRC solutions.

c [mol L^{-1}]	n	A(1598 cm^{-1})
0.228	1.3843	0.295
0.255	1.3957	0.340
0.297	1.4068	0.391
0.347	1.4158	0.448
0.407	1.4279	0.511

0.475	1.4544	0.565
0.543	1.4735	0.631
0.614	1.5149	0.664

Table S5. Concentration-dependent refractive index and IR absorbance of aqueous BTMAPV solutions.

c [mol L^{-1}]	n	A(1640 cm^{-1})
0.267	1.3527	0.063
0.320	1.3575	0.076
0.376	1.3618	0.091
0.457	1.3674	0.110
0.544	1.3741	0.132
0.631	1.3812	0.158
0.766	1.3943	0.189
0.891	1.4072	0.227
1.073	1.4212	0.271
1.267	1.4368	0.326
1.480	1.4577	0.394

Table S6. Concentration-dependent refractive index and IR absorbance of aqueous BTMAPVRC solutions.

c [mol L^{-1}]	n	A(1596 cm^{-1})
0.410	1.3910	0.136
0.477	1.4010	0.173

0.545	1.4140	0.214
0.638	1.4281	0.257
0.727	1.4373	0.310
0.820	1.4647	0.376
1.021	1.4991	0.451

6. Open Circuit Voltage Experiments

The supporting electrolyte was prepared by dissolving NaCl in deionized water to form a 1 M concentration. The 0.1 M TEMPTMA and 0.1 M MV solutions were prepared by either diluting the 50 w/w % TEMPTMA solution or MV powder in the prepared 1 M NaCl supporting electrolyte solution. Volume changes caused by TEMPTMA and MV content were considered to be negligible.

All battery experiments as well as the data acquisition for the Open Circuit Voltage (OCV) cell[5] were conducted in a glovebox using a VSP potentiostat/galvanostat (Bio-Logic, France). The used RFB cells were custom-made flow-through cells using graphite current collectors, GFA-6 felts (SGL, Germany) and Fumasep FAA-3-50 anion exchange membranes (fumatech GmbH, Germany) with an effective membrane area of 5 cm². As OCV cell, a custom-made two compartment electrochemical cell using a Fumasep FAM anion-exchange membrane as a separator and graphite rods as electrodes, was incorporated into electrolyte flow circuit of the non-capacity-limiting compartment. For the OCV sensor calibration, a double fluid circle system was used. A pair of teflon three-way valves was incorporated into the non-capacity-limiting compartment to form two fluid circuits: One, implementing all battery components, namely flow cell, OCV cell, tank; and the second, implementing only flow cell and OCV cell. By means of that it was possible to temporarily establish n-CLC as a limiting compartment and to do cycling in the SOC range from ~0 to ~100%, thereby using full charge-discharge cycles for calibration.

Electrolyte solutions used within the unbalanced compositionally symmetric cell setup were obtained from the 0.1 M TEMPTMA and 0.1 M MV solutions by degassing the solutions via argon bubbling for 40 min and transferring them to the glovebox afterwards. The solutions were used to charge a TEMPTMA/MV cell with a slightly oversized (105%) TEMPTMA half-cell till full capacity (galvanostatic charge with 400 mA current and voltage cutoff at 1.6 V and a subsequent potentiostatic charge at 1.6 V with a current cutoff at 1.25 mA (0.25 mA cm^{-2})) and to mix the generated ~100% SOC 0.1 M MV solution with an equal amount of uncharged 0.1 M MV solution in order to generate a 50% SOC electrolyte. The non-capacity-limiting compartment tank had twice the volume of the capacity-

limiting compartment tank. The same electrolyte composition was used as the reference electrolyte for the OCV cell.

Symmetric cell experiments were conducted with a potentiostatic cycling at 0.2 V in either direction with a current cutoff of 2.5 mA (0.5 mA cm^{-2}). After several electrochemical cycles, the electrolyte was charged till 50% SOC by time restricted galvanostatic charging at 100 mA with 1.6 V voltage cutoff (although the cutoff was never reached). Then the valves were turned and the fluid circuit excluding the non-capacity-limiting compartment tank was studied. Two full electrochemical cycles were measured at the specified conditions for symmetric cell cycling in this experiment, then the electrolyte was again galvanostatically charged till 50% SOC at 100 mA, subsequently the valves were again turned and symmetric cycling was continued with non-capacity-limiting compartment implementing electrolyte from the tank.

The electrochemical data from the OCV cell (see **Figure S1**) was fitted in accordance with Nernst equation for one-electron transfer reaction for anolytes:

$$E = E_0 + \frac{RT}{nF} \cdot ln\left(\frac{1 - \frac{q(t)}{q_{max}}}{\frac{q(t)}{q_{max}}}\right) \tag{5}$$

where E [V] represents the measured OCV potential, E_0 [V] is the reference potential of the reference electrolyte, R [J mol^{-1} K^{-1}] is the gas constant, T [K] is the absolute temperature, n is the number of electrons transferred which is 1 for the MV/MVRC redox pair, F [A s mol^{-1}] is the Faraday constant, and q(t) [A s] is the amount of capacity loaded into the battery at a time t and q_{max} the maximum total amount of charge loaded in the battery during a half-cycle (either total charge capacity or total discharge capacity).

7. UV/Vis Spectroscopy and Association Constant Determination

The UV/Vis experiment were conducted in analogy to the report of Geraskina *et al.*.[16] 0.1 and 0.01 M aqueous MV and BTMAPV solutions were prepared in a glovebox using a 0.1 M pH 8 phosphate buffer. A sufficient amount of sodium dithionate stock solution (prepared in the same buffer solution) was subsequently added in order to be present in an excess to completely reduce the viologens to their respective radical cations. The reduced solutions were subsequently diluted with the buffer solution (pH 8, phosphate) to yield viologen concentrations of 20, 50, 100, 200, 500 and 1000 µM, respectively. The final concentration of sodium dithionate in all UV/Vis solutions was 2 mM.

Prior to the UV/Vis measurements, a reference spectrum from a solution containing 2 mM sodium dithionite in the buffer solution was collected to minimize the signal stemming from the buffer solution and glass cuvette. The UV/Vis spectra of the prepared solutions were measured on a LAMBDA 750 UV/Vis/NIR spectrometer from PerkinElmer (Waltham, Massachusetts). The spectra were recorded between 400 and 1200 nm with a resolution of 4 nm. During the measurement, the lamp and monochromator were switched at 860 nm to achieve maximum sensitivity.

The association constant was determined using a non-linear fitting procedure.[17,18] For this procedure, the absorbance at 864 nm was fitted, as it stems from viologen radical cation dimers[16] using the following equation

$$A = \frac{C}{8K_a}\left(1 + 4K_a c_{tot} - \sqrt{1 + 8K_a c_{tot}}\right) \tag{6}$$

Where *A* is the absorbance, *C* a fitting parameter, K_a the association constant and c_{tot} the total viologen concentration. The equation can be derived using the law of mass action and the mass balance according to

$$K_a = \frac{[dimer]}{[monomer]^2} \tag{7}$$

$$c_{tot} = [monomer] - 2 \cdot [dimer] \tag{8}$$

The recorded UV/Vis spectra and the corresponding non-linear fits can be seen in **Figure S26** and **Figure S27**.

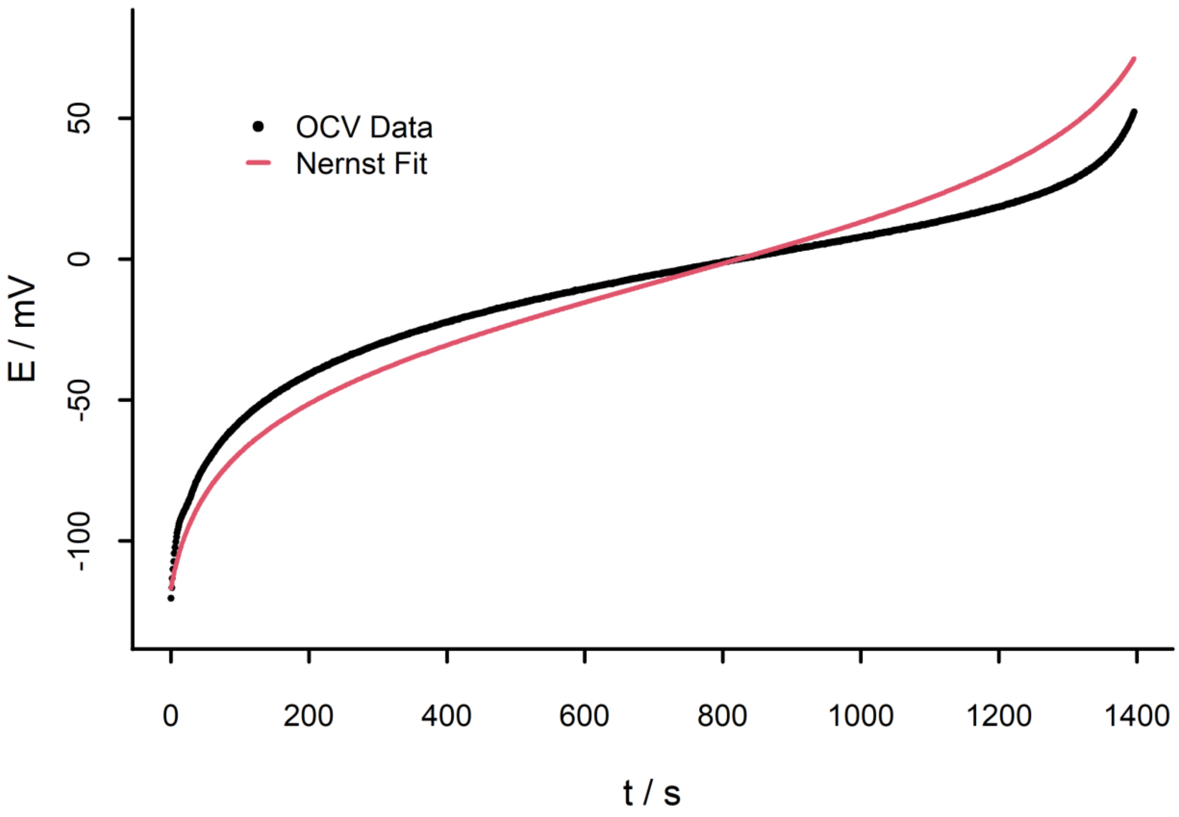

Figure S1. OCV data recorded during galvanostatic charging of a MV/MVRC RFB half cell (black dots). The experimental data deviates significantly from the ideal Nernst curve (red line).

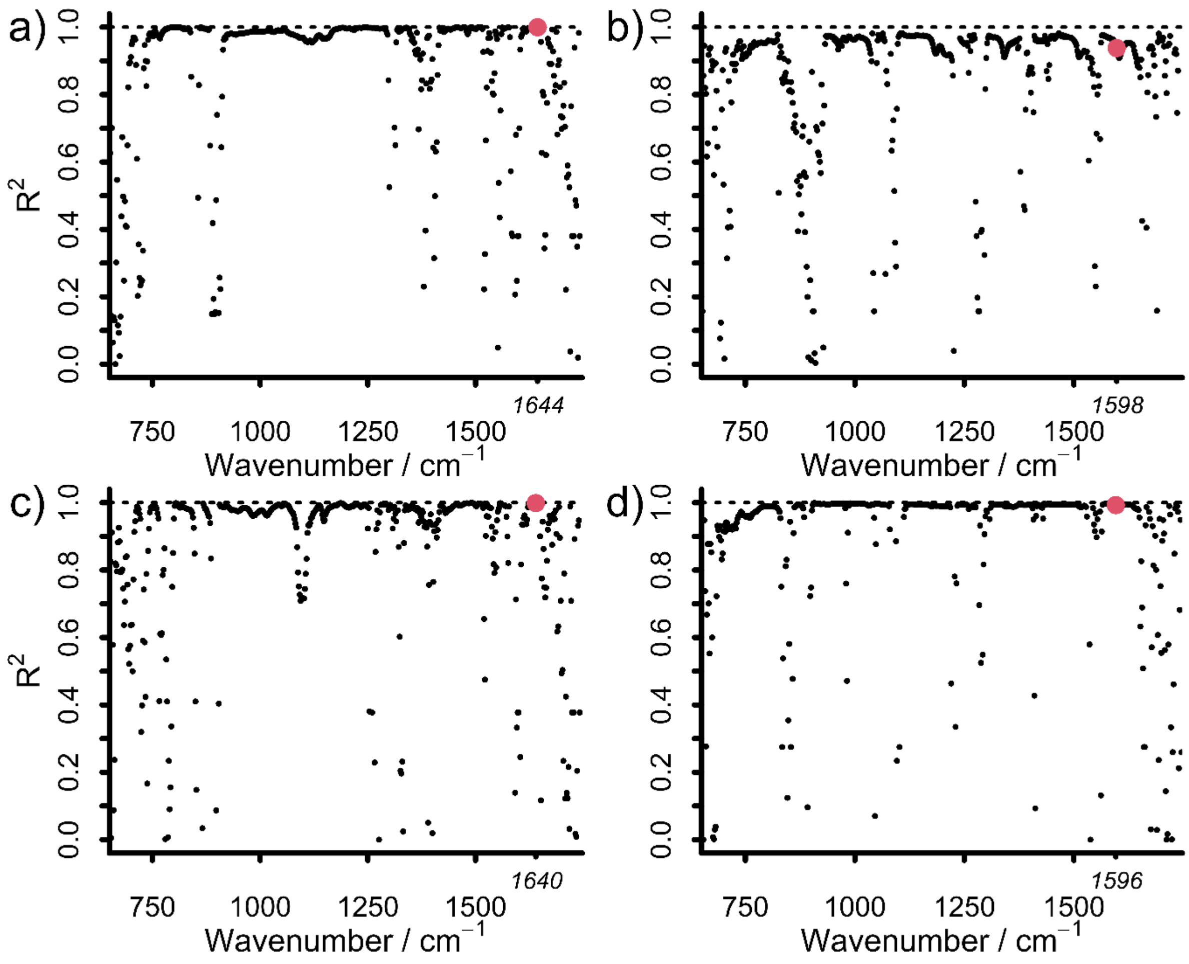

Figure S2. R^2 values received when performing a linear regression between the IR absorbance at a given wavenumber position and the concentration of MV (a), MVRC (b), BTMAPV (c) and BTMAPVRC (d). The red dot highlights the wavenumber position chosen for the following Lambert Beer derived analysis (see **Error! Reference source not found.** and **Error! Reference source not found.**). As can be seen, most wavenumber positions are reasonable choices to derive the electrolytes' concentration from its IR absorbance, as most R^2 values are close to 1.

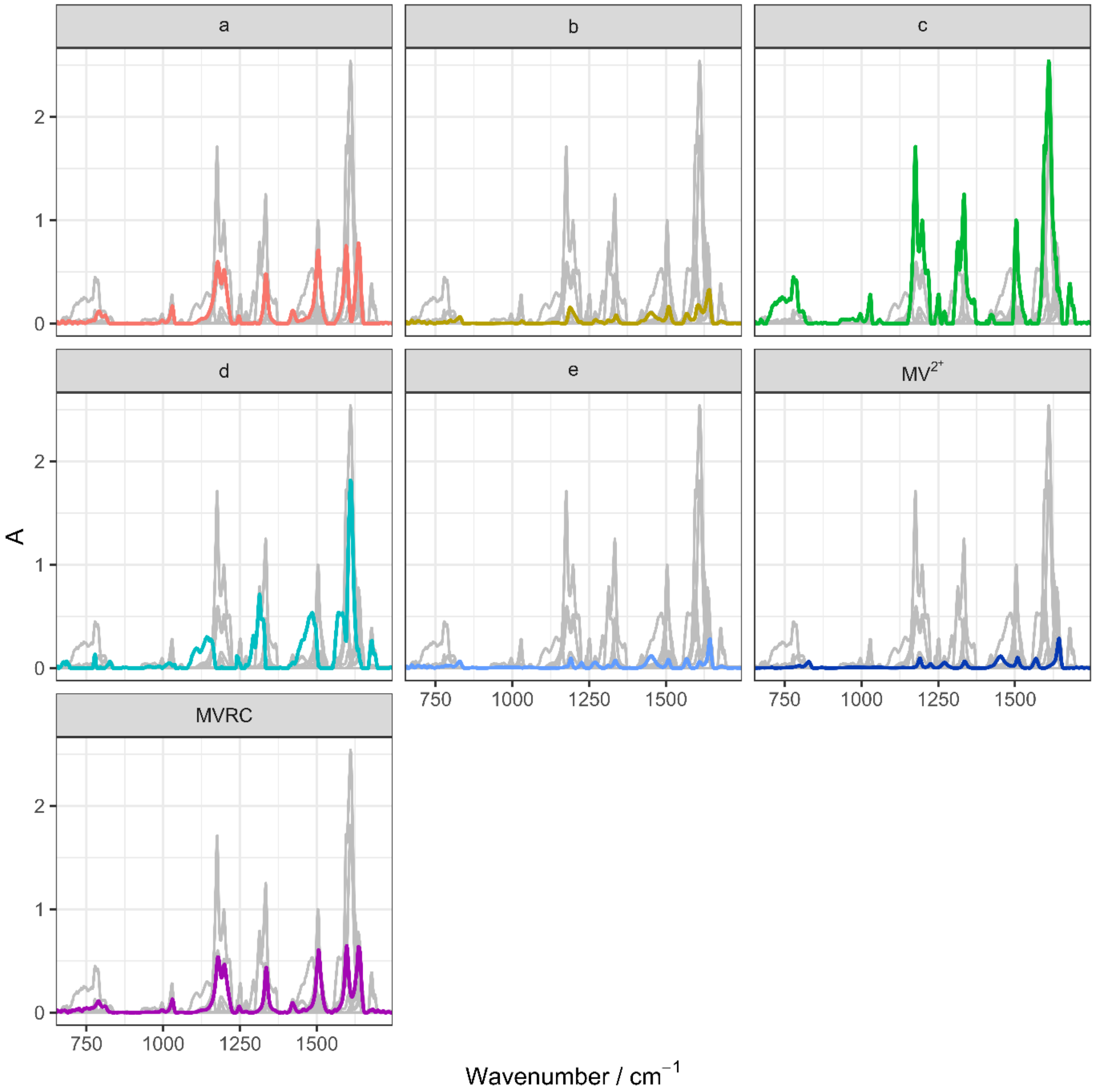

Figure S3. IR spectra extracted by MCR-ALS/NNLS algorithms using five components as well as the IR spectra of MV and MVRC as references. The spectra are the same also shown in **Error! Reference source not found.**a. Each panel highlights the IR spectrum of one species (colored) in reference to all other IR spectra (grey).

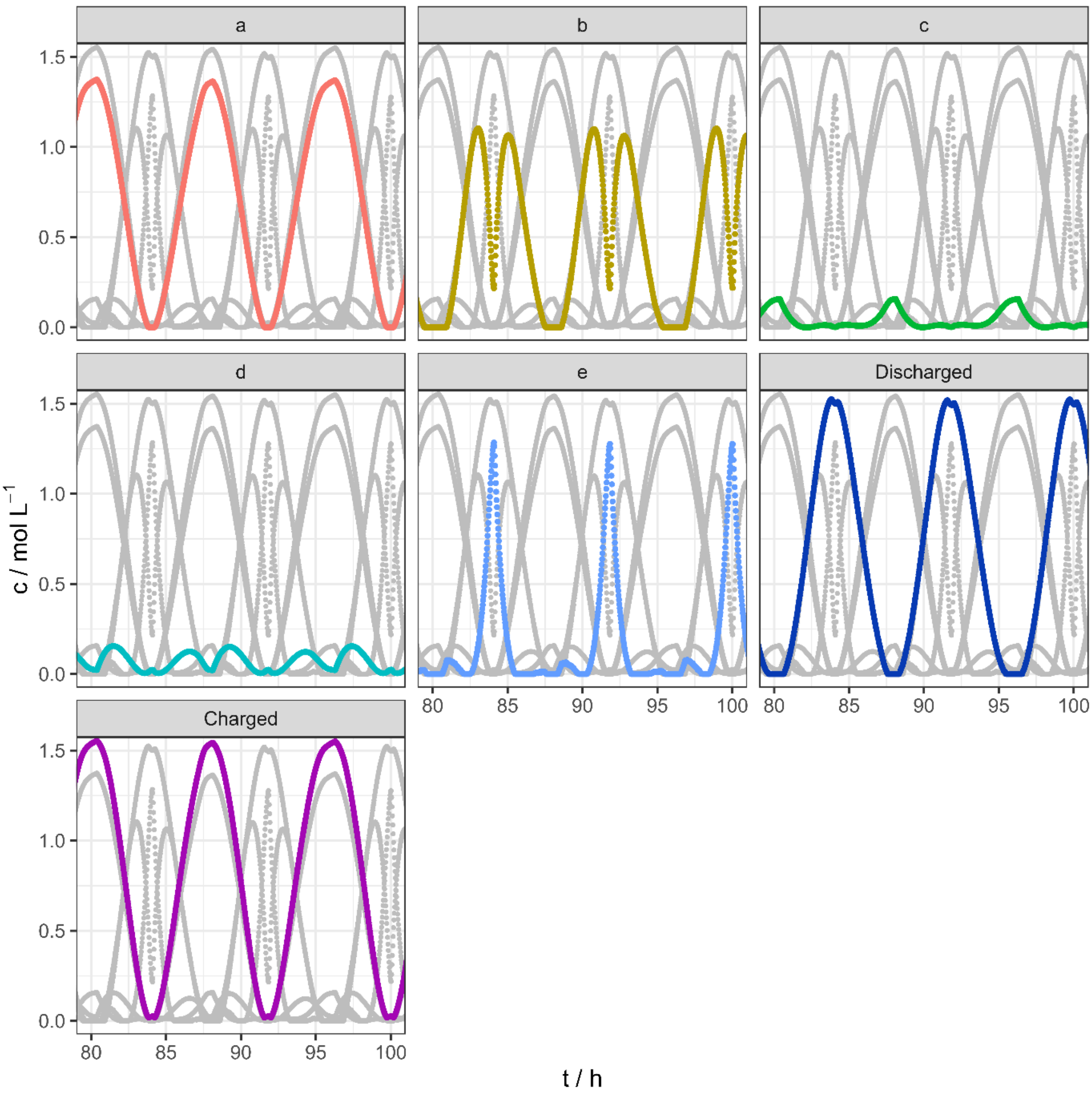

Figure S4. Concentration profiles extracted by MCR-ALS/NNLS algorithms using five components as well as the cumulative concentration of all charged and discharged species for a MV RFB. The concentration profiles are the same also shown in **Error! Reference source not found.**b. Each panel highlights the time-dependent concentration of one species (colored) in reference to all other concentrations (grey).

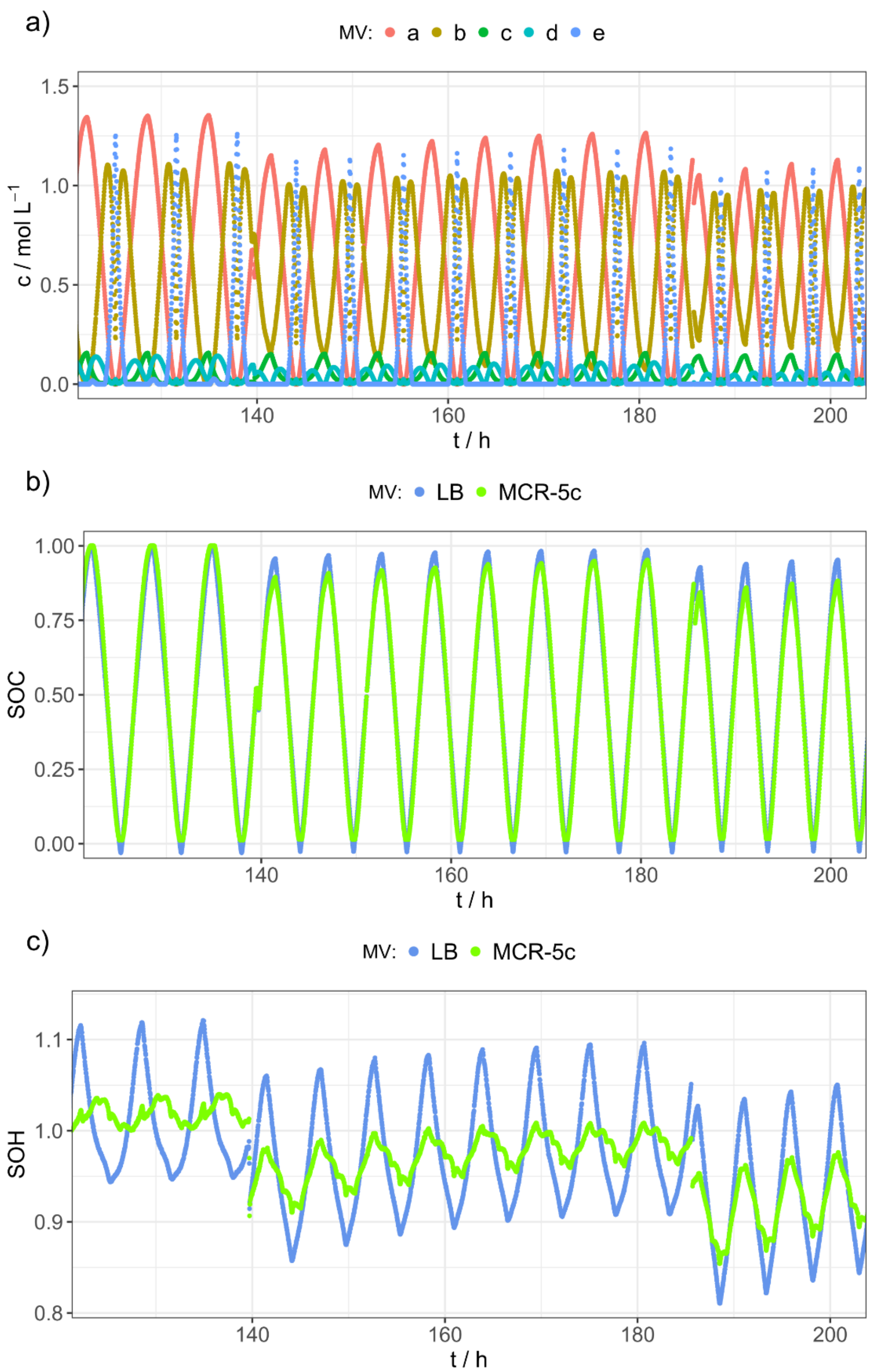

Figure S5. Concentration profiles (a), SOC (b) and SOH values (c) extracted by MCR-ALS/NNLS algorithms using five components on a MV RFB. The data shown covers a longer

time period compared to **Error! Reference source not found.** and also features the two artificial SOH changes after 140 and 185 h which are highlighted in **Error! Reference source not found.**.

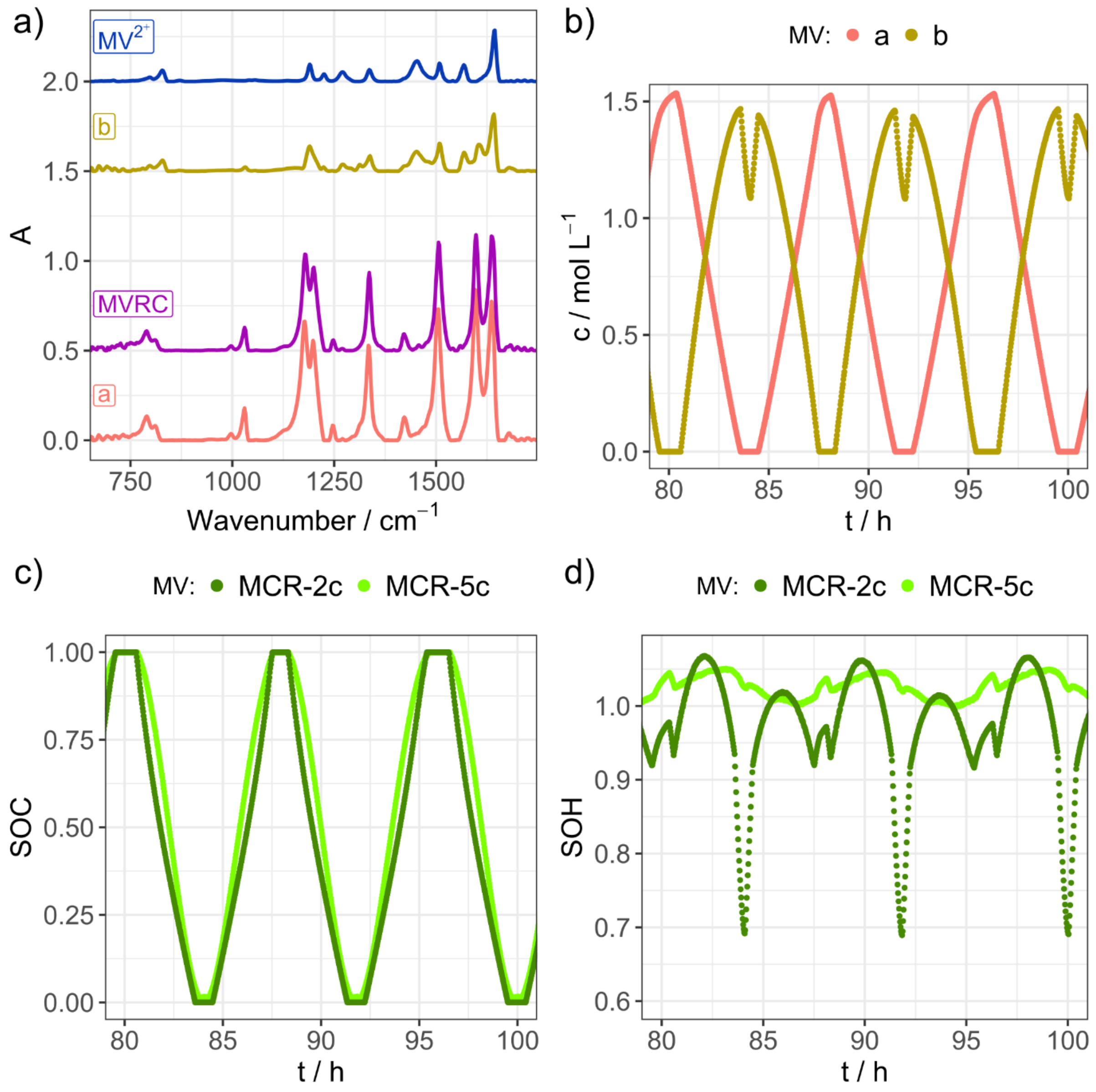

Figure S6. IR spectra (a), concentration profiles (b), SOC (c) and SOH values (d) extracted by MCR-ALS/NNLS algorithms using two components for a MV RFB. The SOC and SOH values are compared to the values extracted by MCR-ALS/NNLS algorithms using five components. The SOH value varies significantly when the SOC is low.

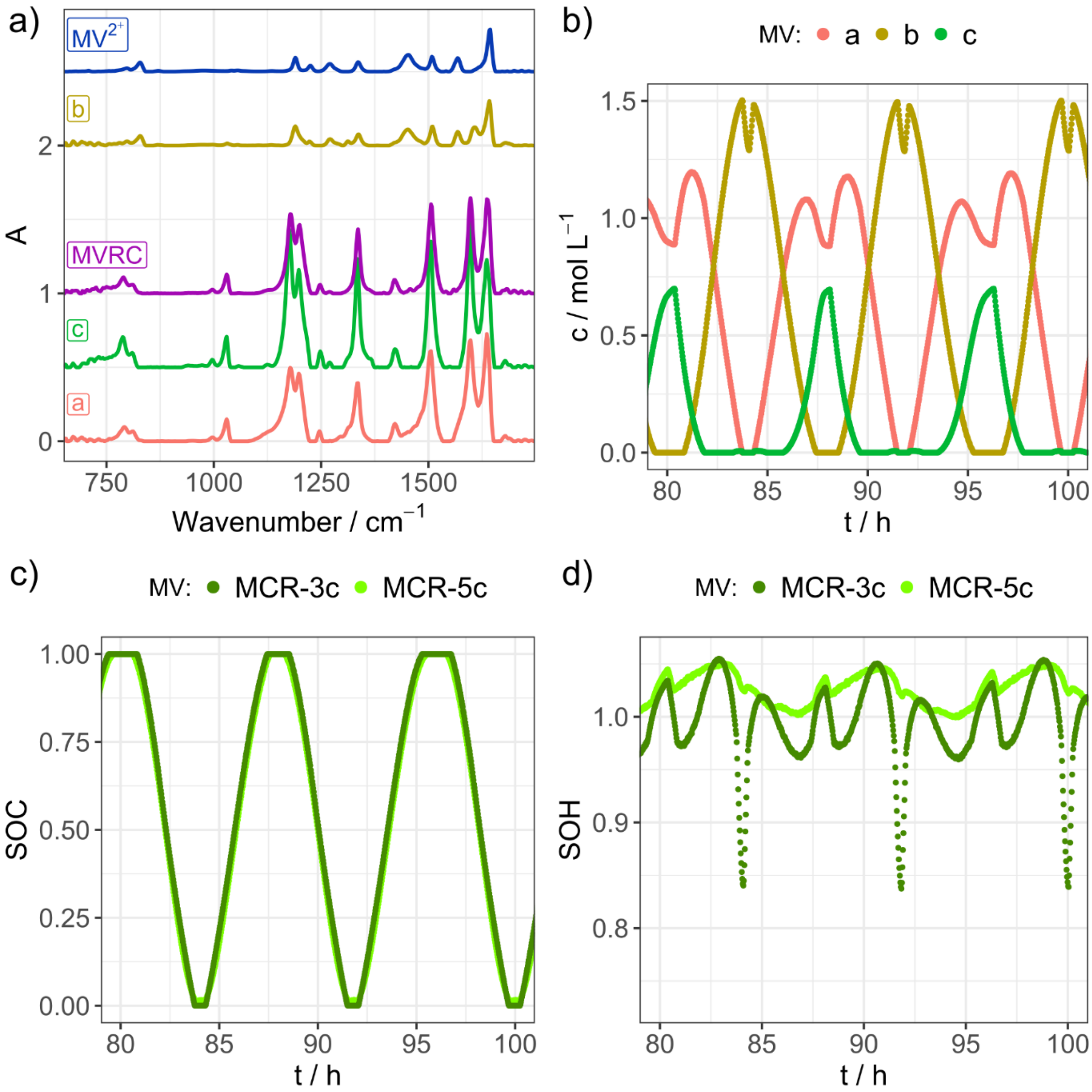

Figure S7. IR spectra (a), concentration profiles (b), SOC (c) and SOH values (d) extracted by MCR-ALS/NNLS algorithms using three components for a MV RFB. The SOC and SOH values are compared to the values extracted by MCR-ALS/NNLS algorithms using five components. The SOH value varies significantly when the SOC is low.

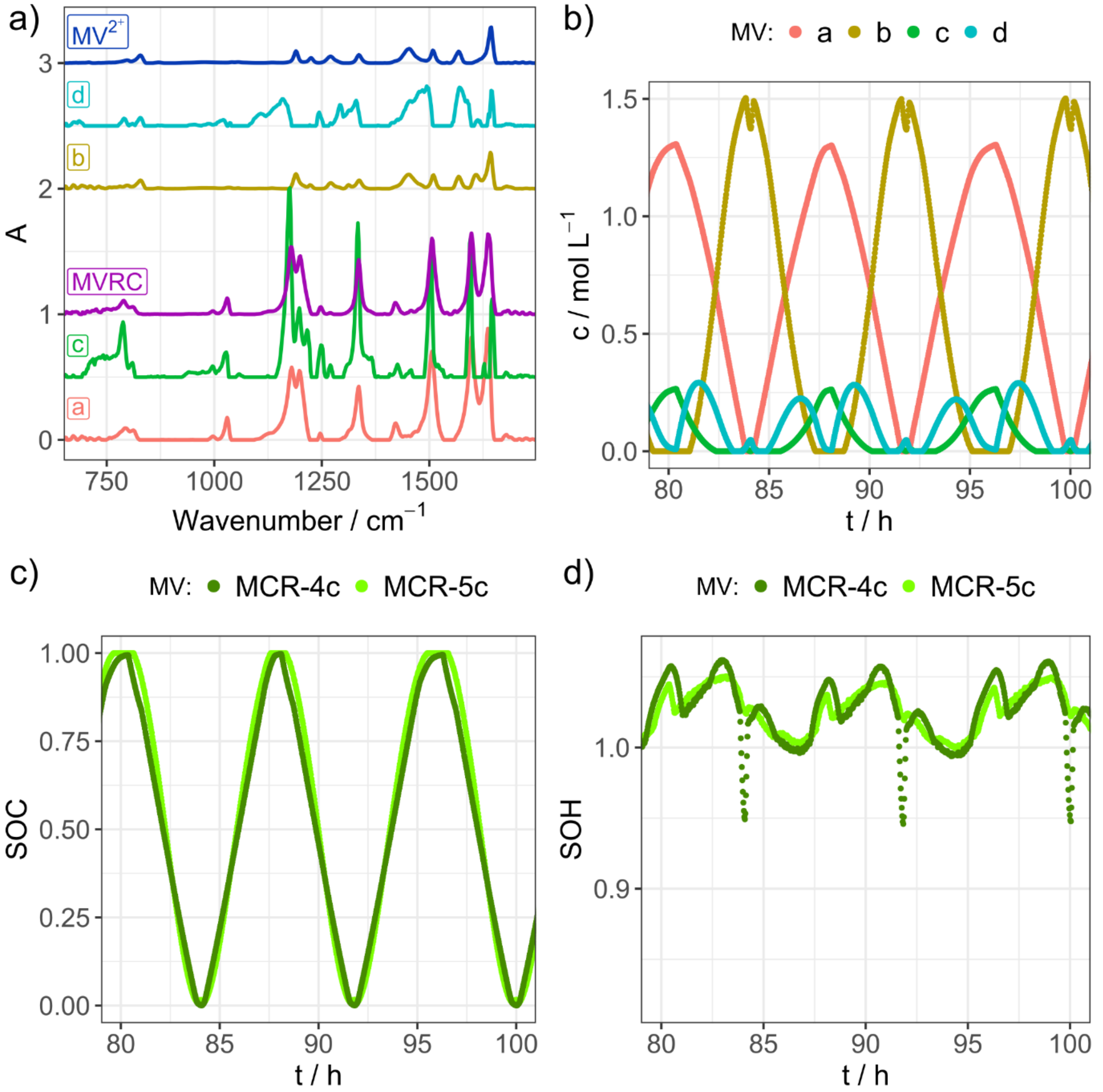

Figure S8. IR spectra (a), concentration profiles (b), SOC (c) and SOH values (d) extracted by MCR-ALS/NNLS algorithms using four components for a MV RFB. The SOC and SOH values are compared to the values extracted by MCR-ALS/NNLS algorithms using five components. The SOH value varies significantly when the SOC is low.

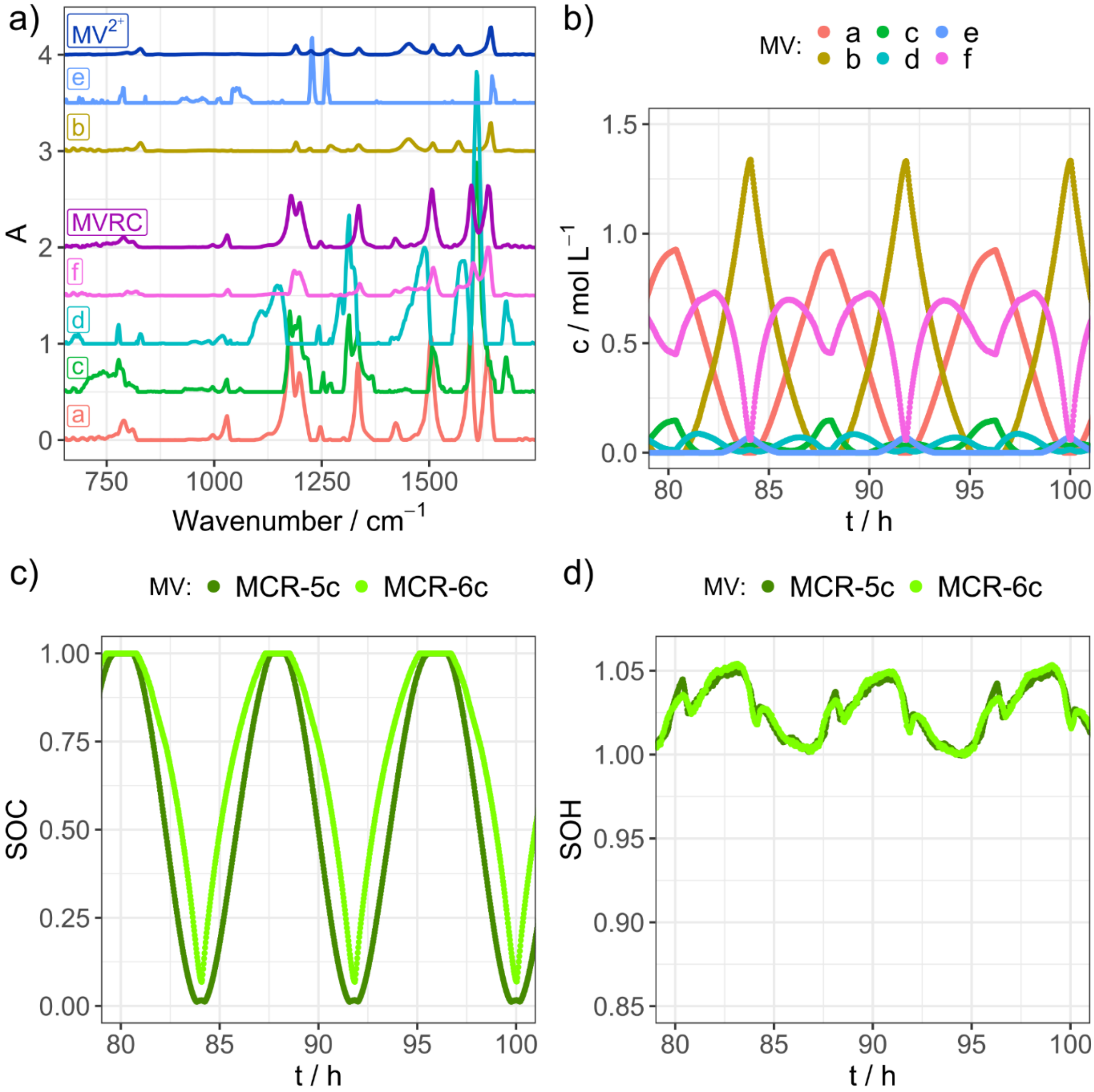

Figure S9. IR spectra (a), concentration profiles (b), SOC (c) and SOH values (d) extracted by MCR-ALS/NNLS algorithms using six components for a MV RFB. The SOC and SOH values are compared to the values extracted by MCR-ALS/NNLS algorithms using five

components. The SOC values deviate from the expected linearity. The accuracy of the SOH determination is not improved by using six instead of just five components.

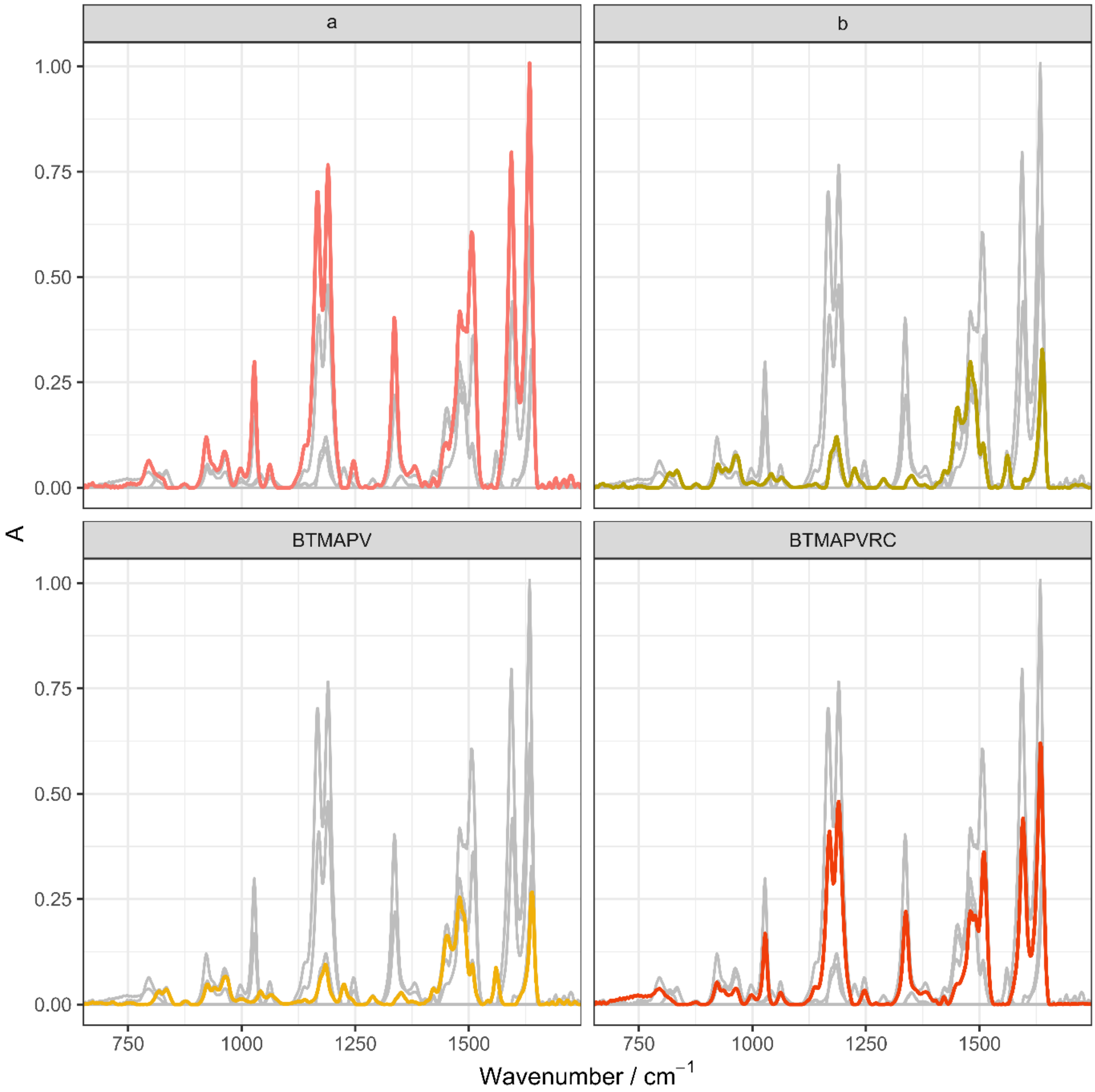

Figure S10. IR spectra extracted by MCR-ALS/NNLS algorithms using two components as well as the IR spectra of BTMAPV and BTMAPVRC as references. The spectra are the same

also shown in **Error! Reference source not found.**a. Each panel highlights the IR spectrum of one species (colored) in reference to all other IR spectra (grey).

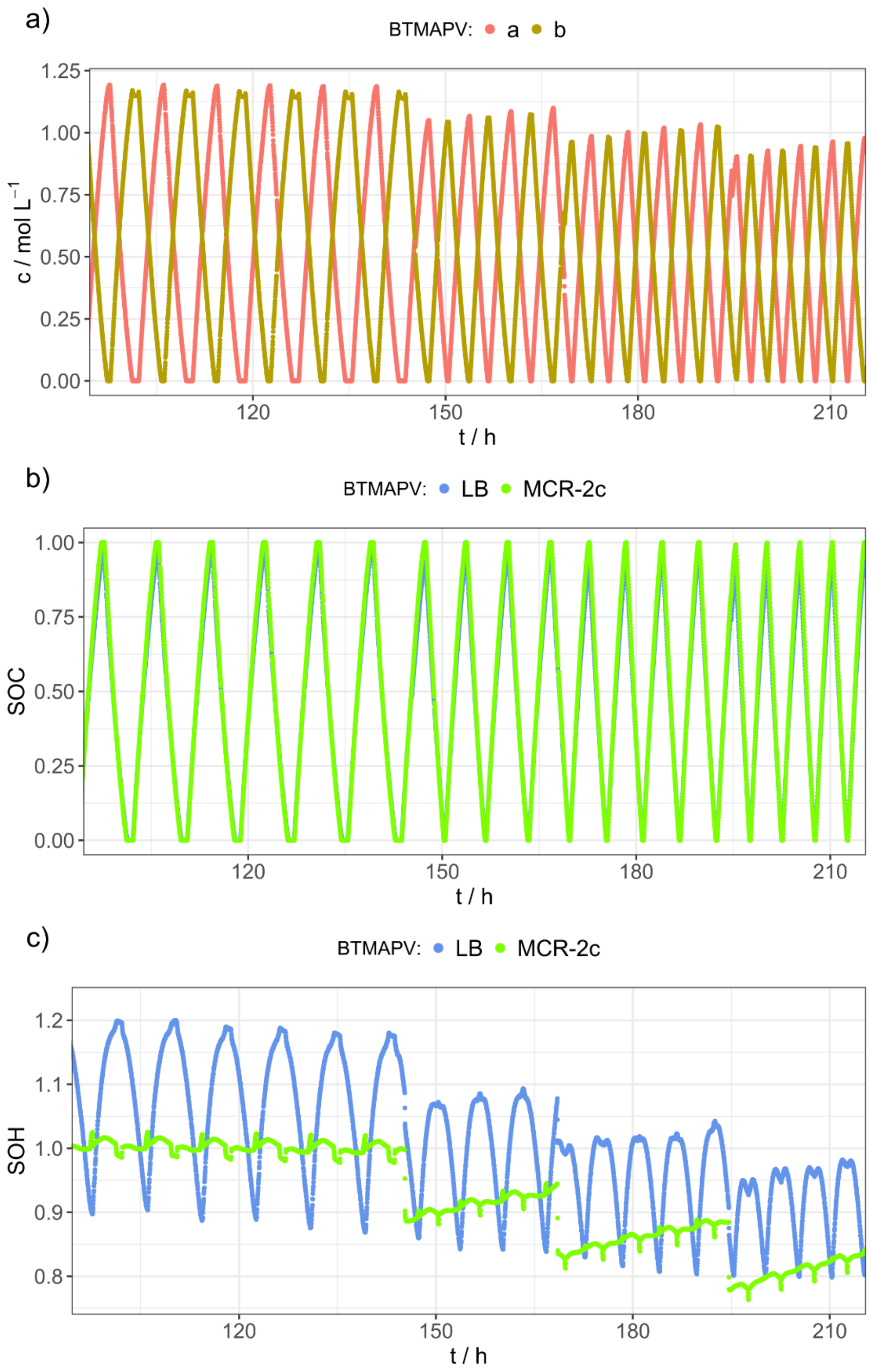

Figure S11. Concentration profiles (a), SOC (b) and SOH values (c) extracted by MCR-ALS/NNLS algorithms using two components for a BTMAPV RFB. The data shown covers a longer time period compared to Error! Reference source not found. and also features the three

artificial SOH changes after 145, 162 and 190 h which are highlighted in **Error! Reference source not found.**.

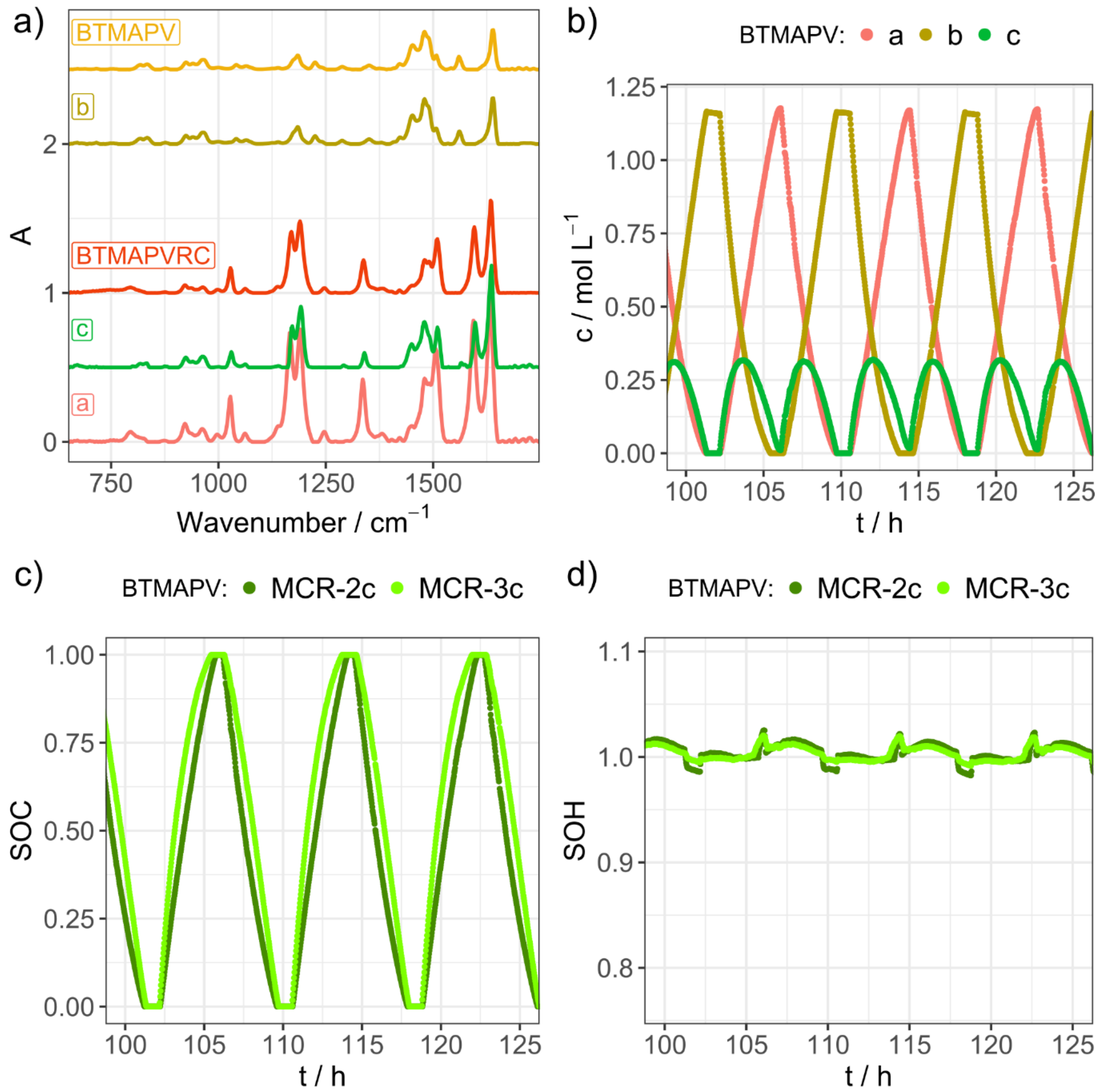

Figure S12. IR spectra (a), concentration profiles (b), SOC (c) and SOH values (d) extracted by MCR-ALS/NNLS algorithms using three components for a BTMAPV RFB. The SOC and SOH values are compared to the values extracted by MCR-ALS/NNLS algorithms using two components. The SOH value varies significantly when the SOC is low.

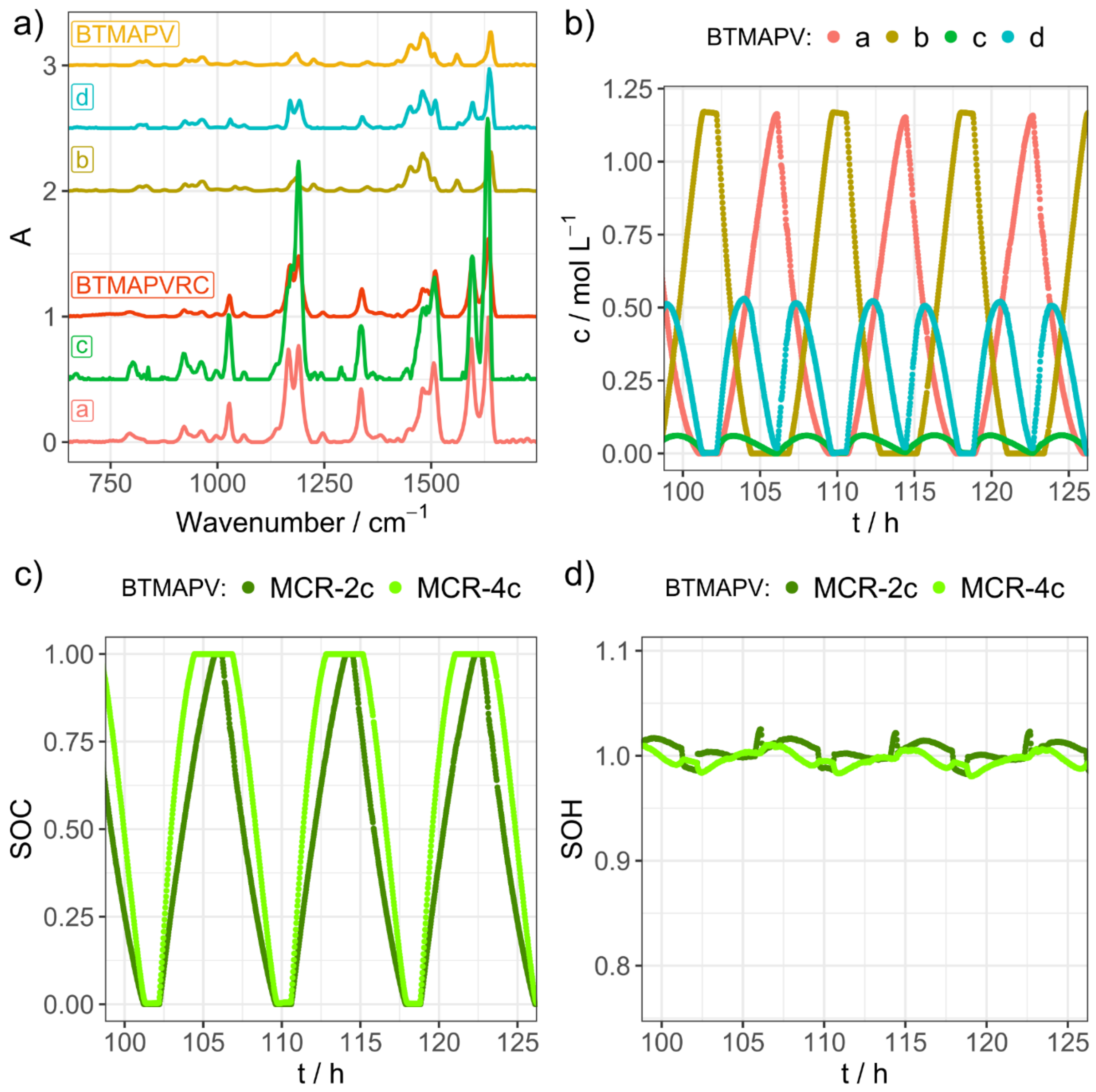

Figure S13. IR spectra (a), concentration profiles (b), SOC (c) and SOH values (d) extracted by MCR-ALS/NNLS algorithms using four components for a BTMAPV RFB. The SOC and SOH values are compared to the values extracted by MCR-ALS/NNLS algorithms using two components.

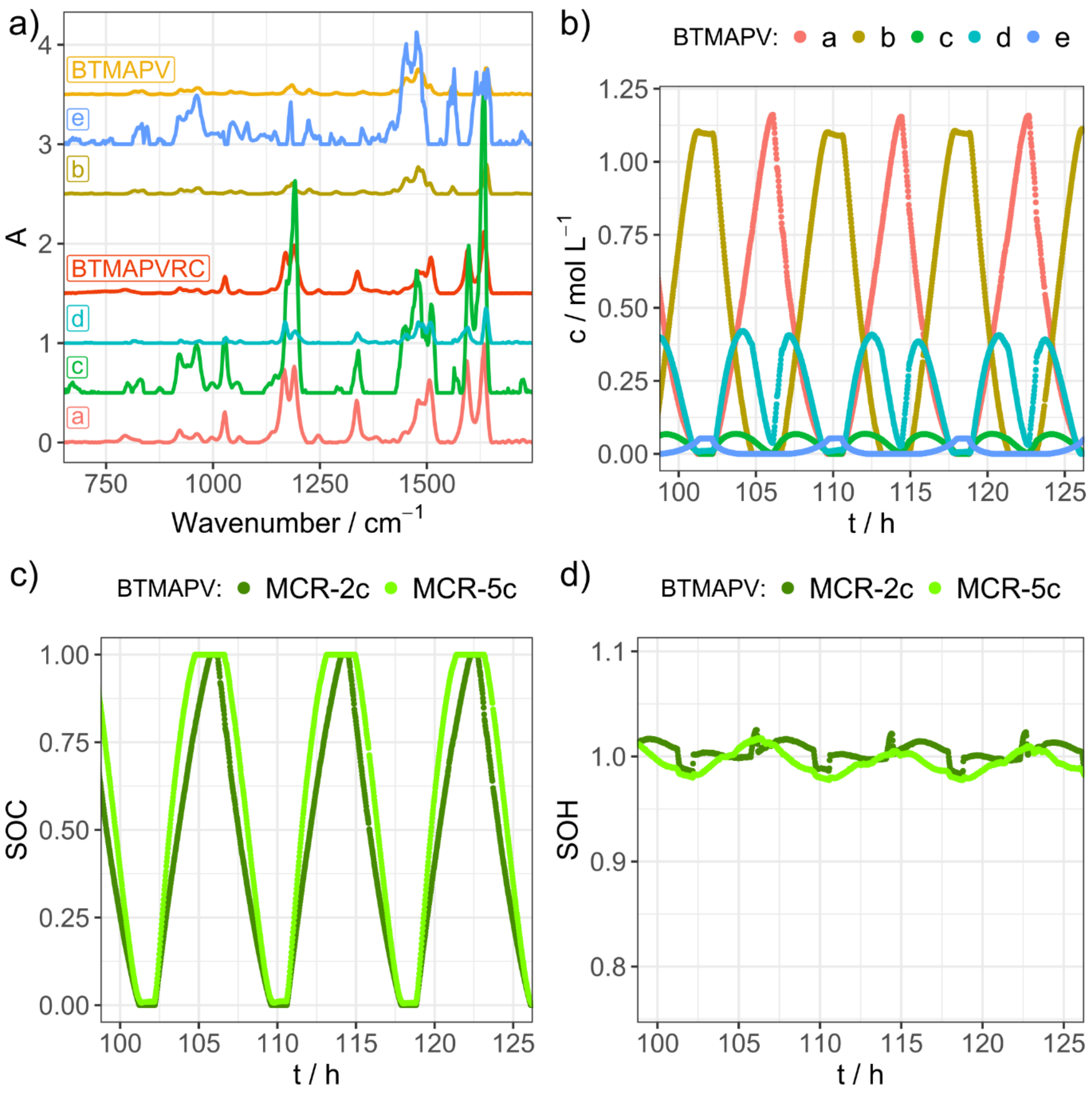

Figure S14. IR spectra (a), concentration profiles (b), SOC (c) and SOH values (d) extracted by MCR-ALS/NNLS algorithms using five components for a BTMAPV RFB. The SOC and SOH values are compared to the values extracted by MCR-ALS/NNLS algorithms using two components.

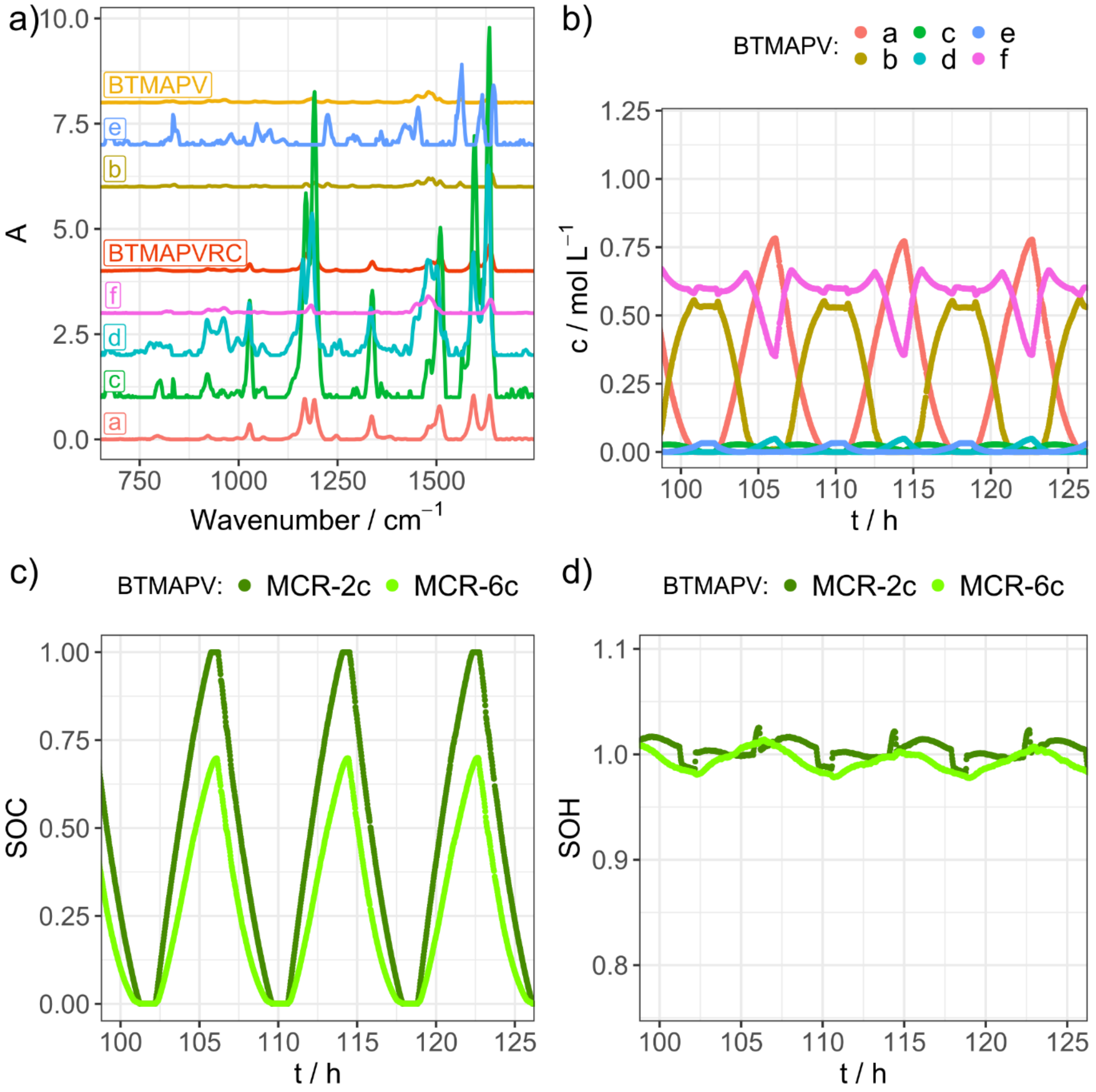

Figure S15. IR spectra (a), concentration profiles (b), SOC (c) and SOH values (d) extracted by MCR-ALS/NNLS algorithms using six components for a BTMAPV RFB. The SOC and SOH values are compared to the values extracted by MCR-ALS/NNLS algorithms using two components. The SOC values deviate from the expected linearity. The accuracy of the SOH determination is not improved by using six instead of just two components.

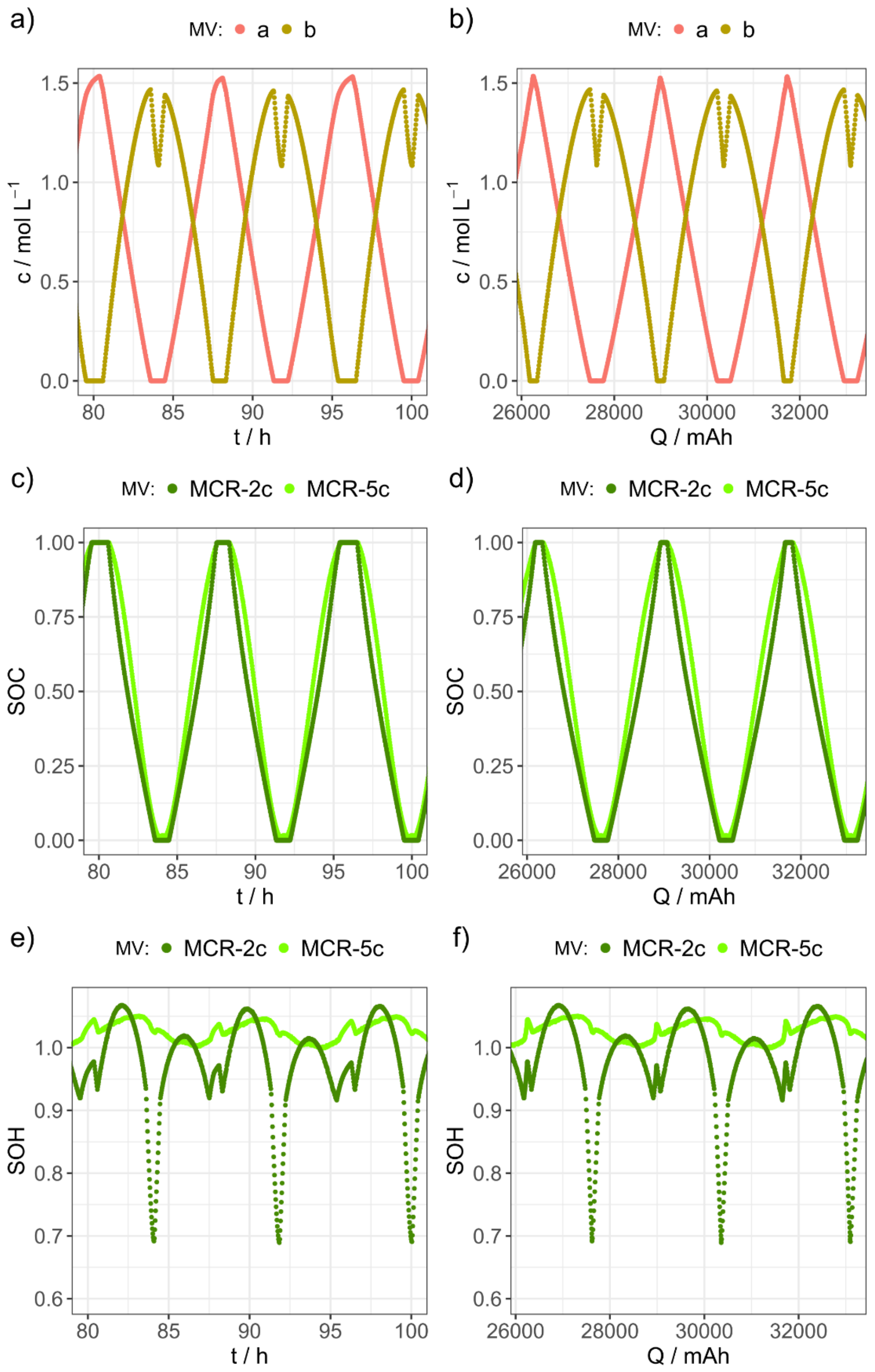

Figure S16. Concentration profiles (a, b), SOC (c, d) and SOH values (e, f) extracted by MCR-ALS/NNLS algorithms using two components for a MV RFB plotted against time (a, c, e) and against the total transferred charge (b, d, f). In neither case the concentration profiles are linear indicating that more than 2 components are involved in the charging process.

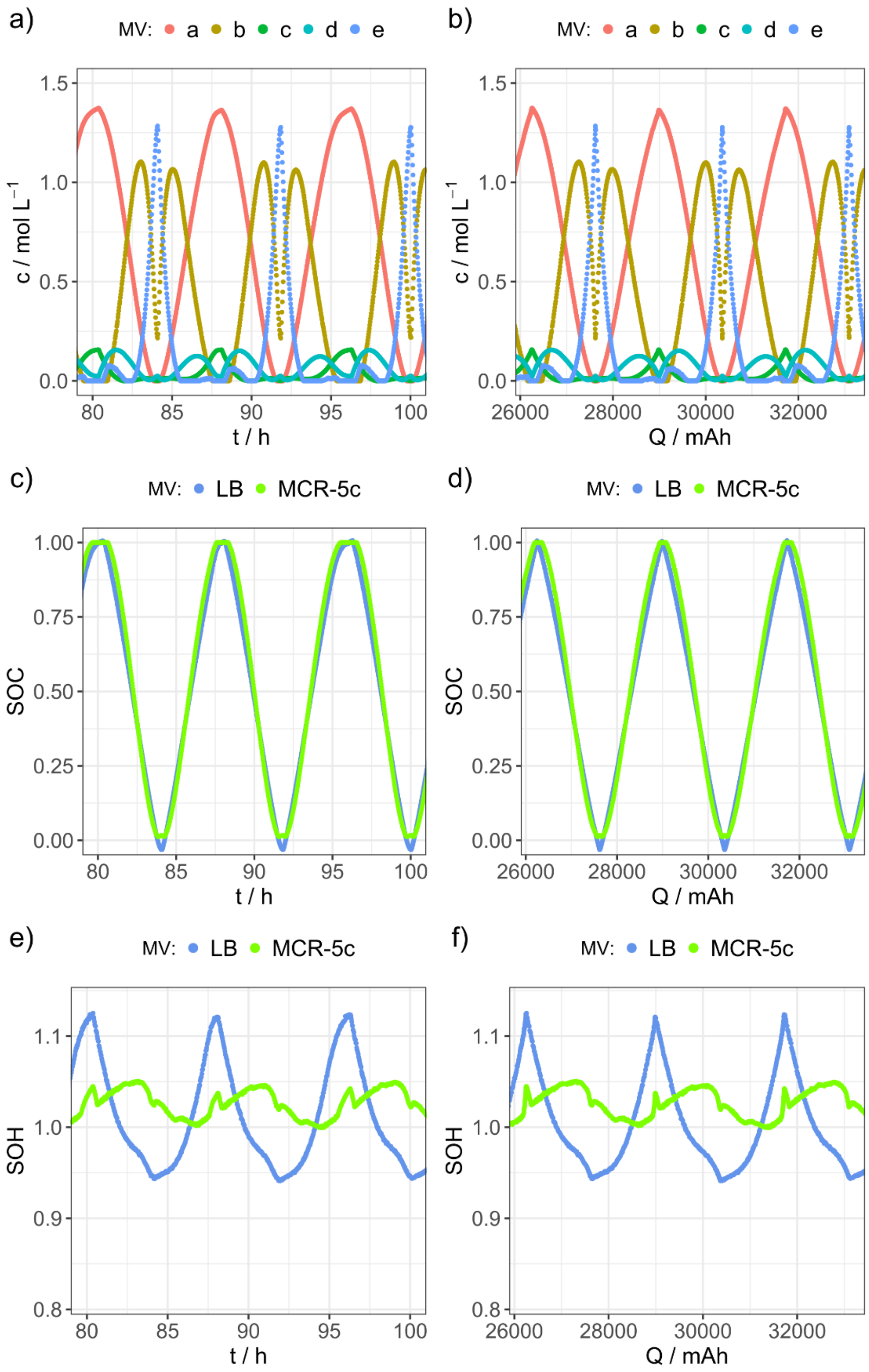

Figure S17. Concentration profiles (a, b), SOC (c, d) and SOH values (e, f) extracted by MCR-ALS/NNLS algorithms using five components for a MV RFB plotted against time (a, c, e) and against the total transferred charge (b, d, f).

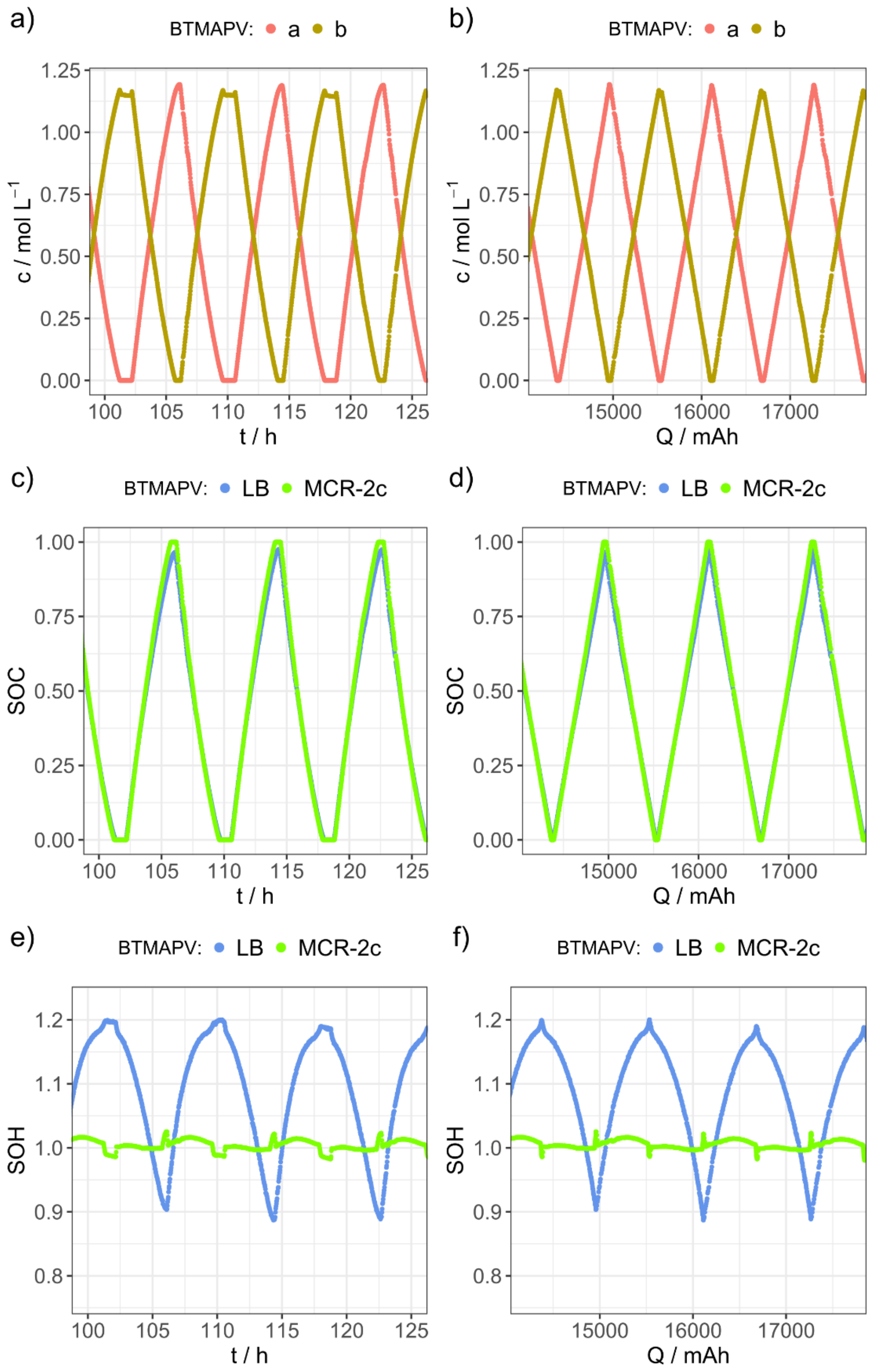

Figure S18. Concentration profiles (a, b), SOC (c, d) and SOH values (e, f) extracted by MCR-ALS/NNLS algorithms using two components for a BTMAPV RFB plotted against time (a, c, e) and against the total transferred charge (b, d, f). The plot against time shows a

non-linear relation which stems from the galvanostatic charging process. Following Faradays law concentration and SOC change linearly when the total transferred charge is used as x axis.

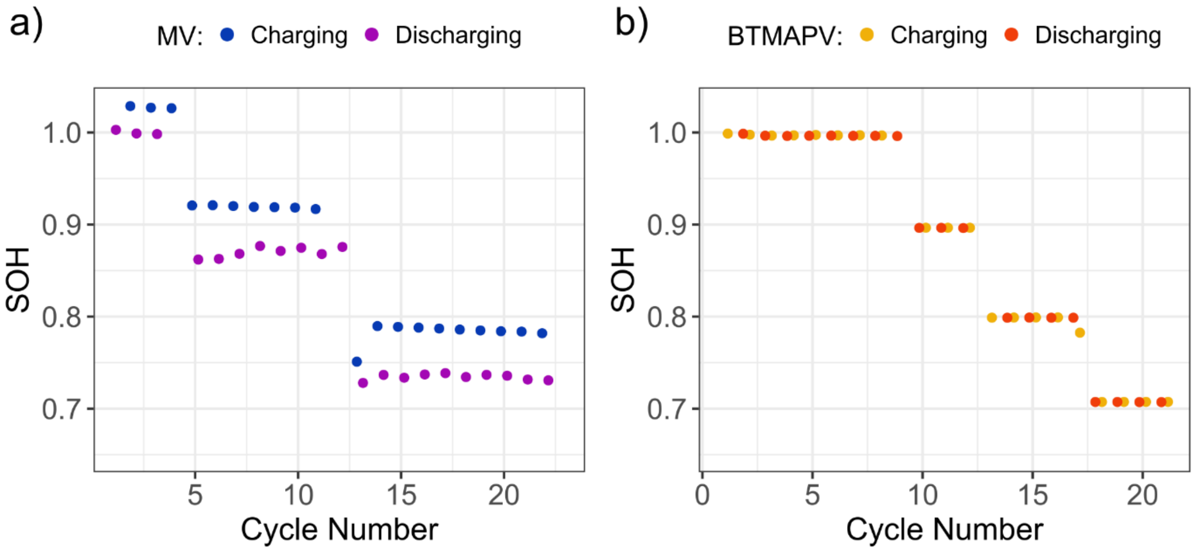

Figure S19. a) SOH derived by Coulomb counting (CC) for a MV RFB averaged over charging (blue) and discharging half cycles (purple). b) CC derived SOH for a BTMAPV RFB averaged over charging (yellow) and discharging half cycles (red). The SOH is artificially lowered by 10% in both cases multiple times.

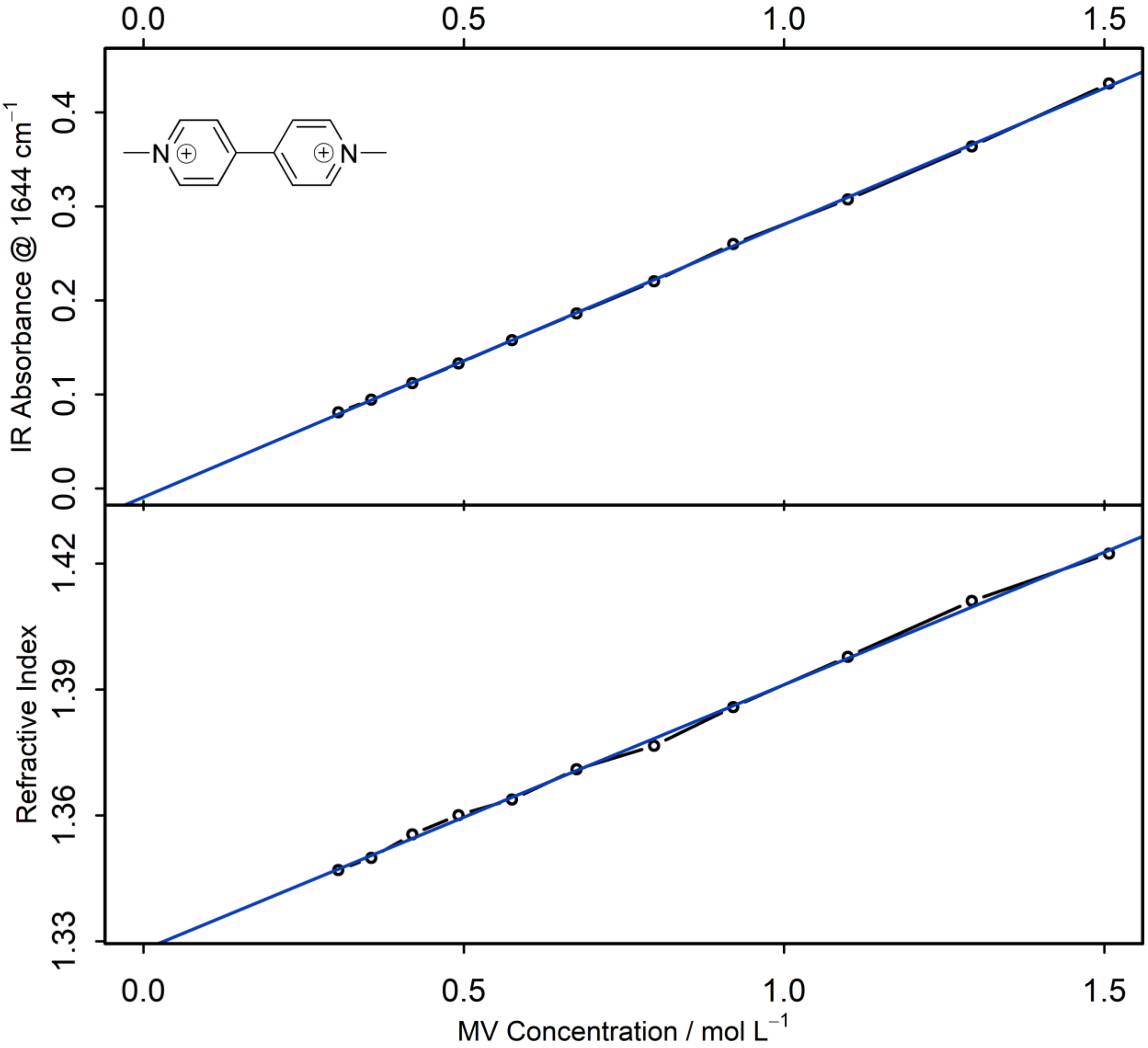

Figure S20. Calibration curves between the concentration of an aqueous MV solution and its IR absorbance at 1644 cm^{-1} (top) as well as its refractive index (bottom). All properties scale linearly with the MV concentration.

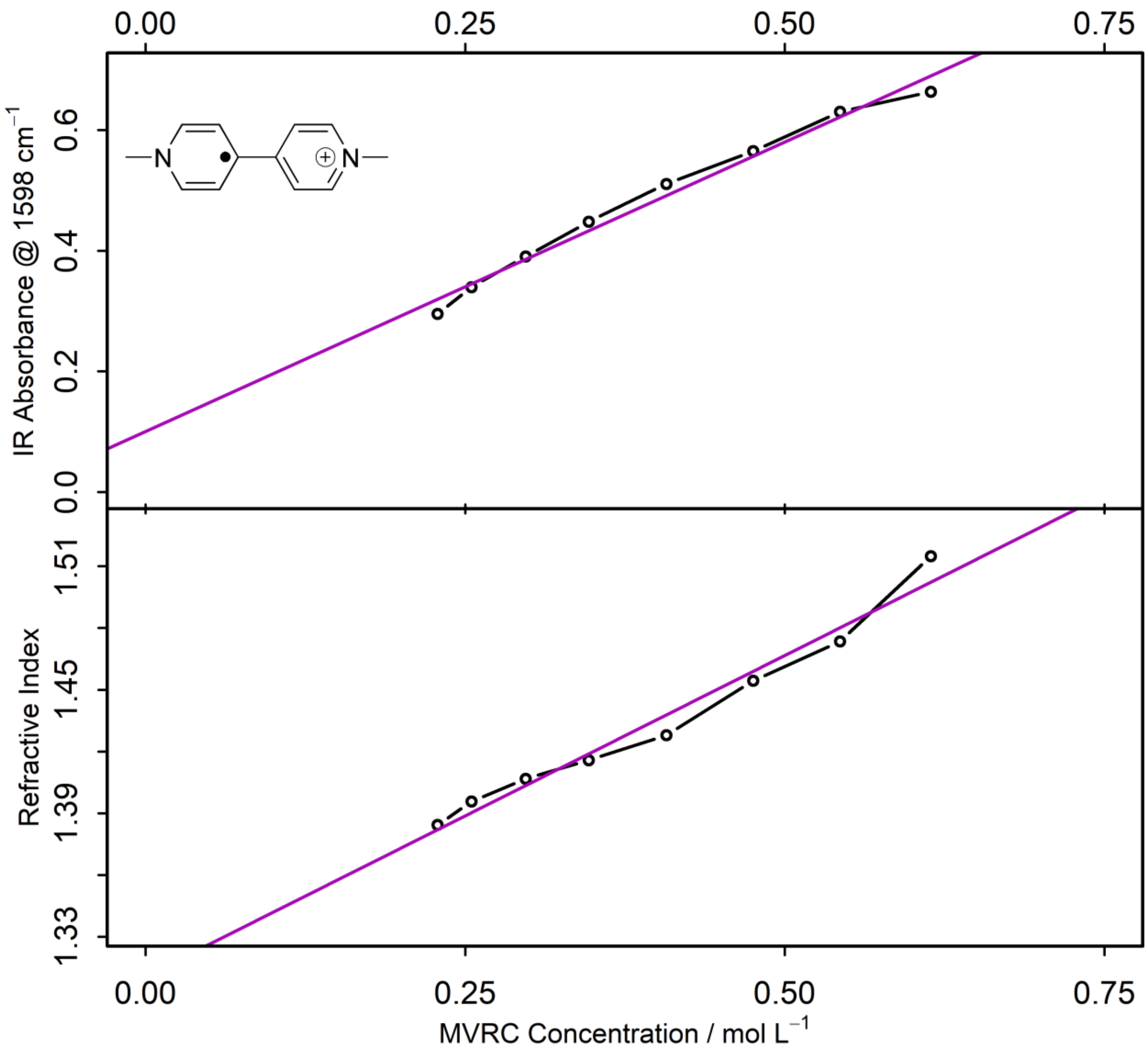

Figure S21. Calibration curves between the concentration of an aqueous MVRC solution and its IR absorbance at 1598 cm^{-1} (top) as well as its refractive index (bottom). All properties scale linearly with the MVRC concentration.

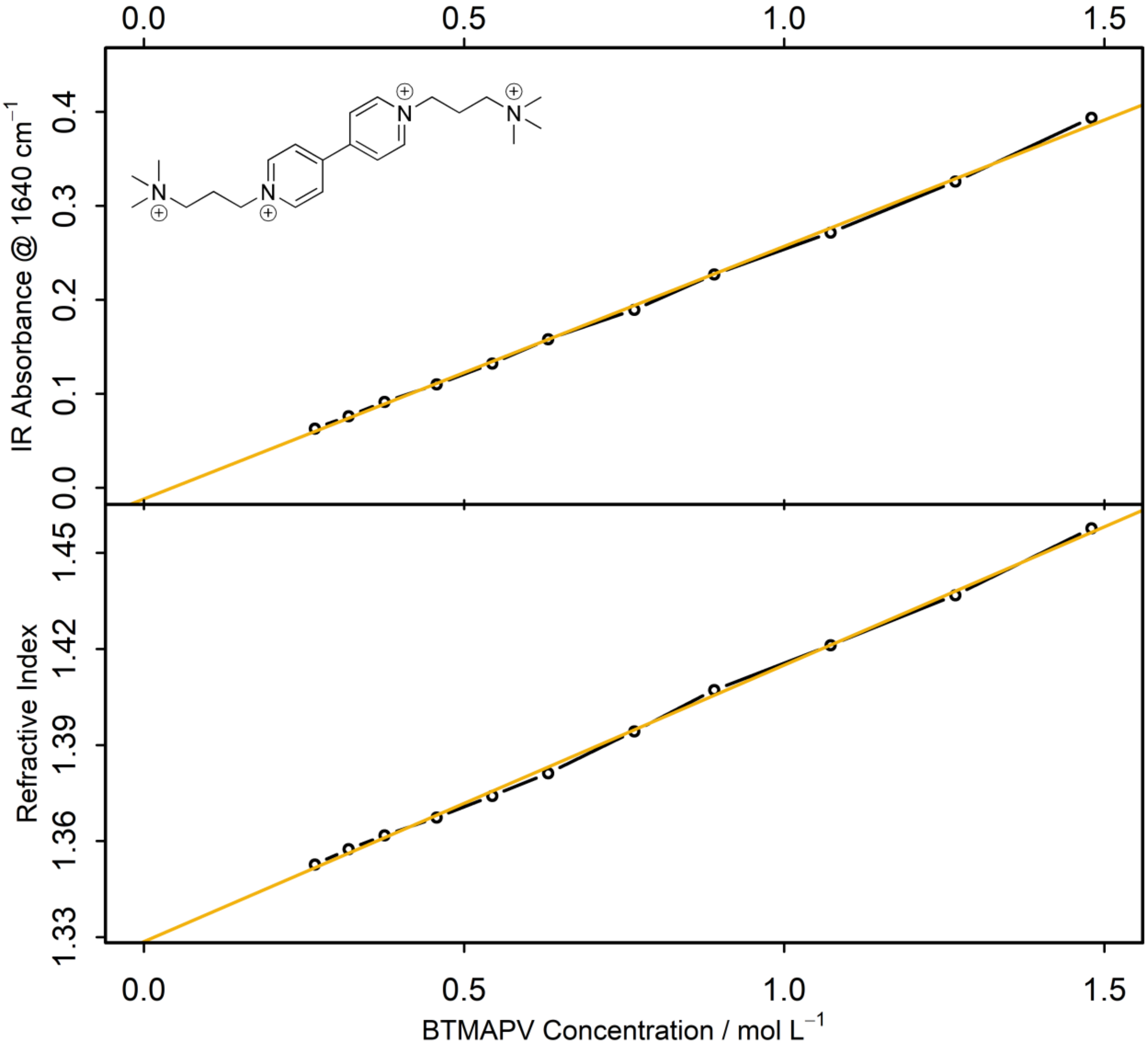

Figure S22. Calibration curves between the concentration of an aqueous BTMAPV solution and its IR absorbance at 1640 cm^{-1} (top) as well as its refractive index (bottom). All properties scale linearly with the BTMAPV concentration.

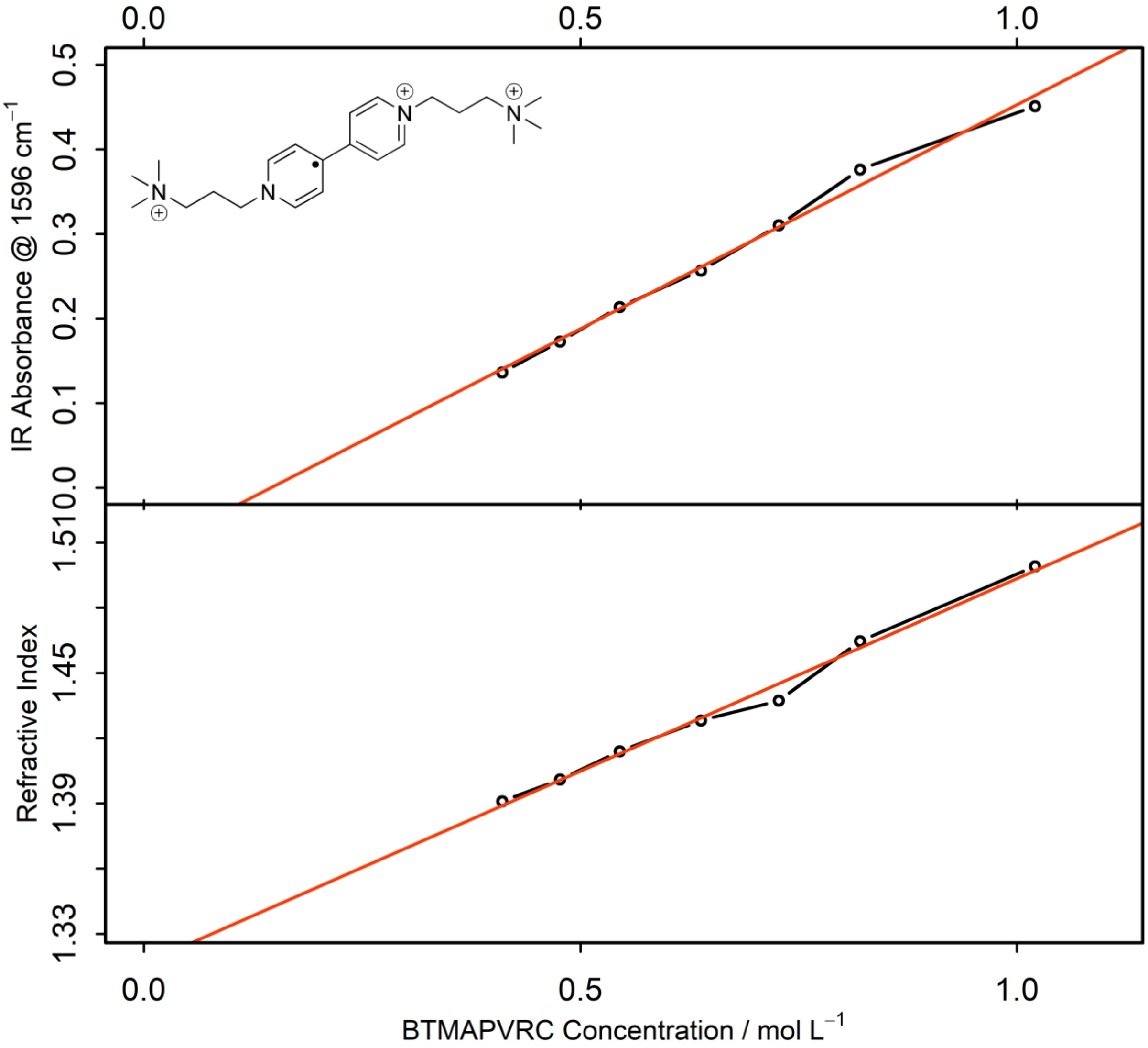

Figure S23. Calibration curves between the concentration of an aqueous BTMAPVRC solution and its IR absorbance at 1596 cm^{-1} (top) as well as its refractive index (bottom). All properties scale linearly with the BTMAPVRC concentration.

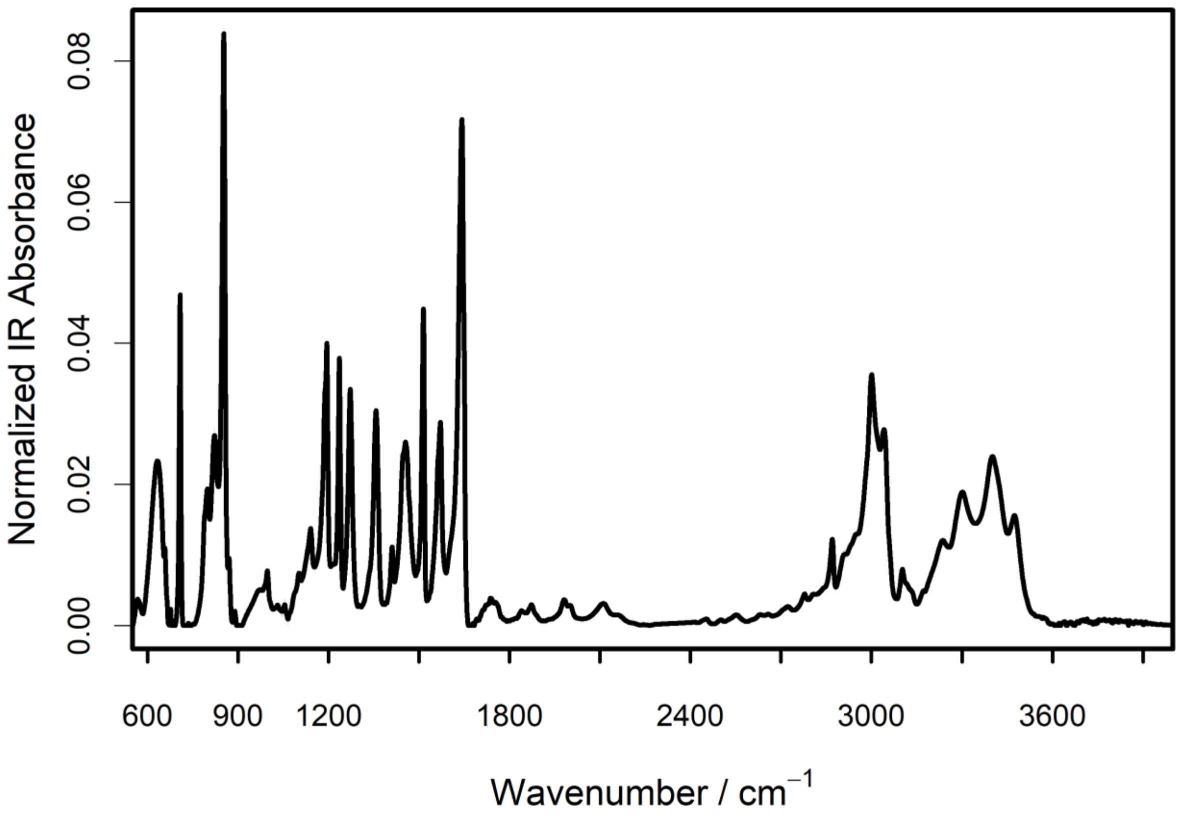

Figure S24. Baseline corrected and normalized ATR IR spectrum of MV crystals. Band positions: 633, 708, 799, 822, 853, 997, 1141, 1195, 1236, 1272, 1358, 1411, 1456, 1515, 1571, 1643, 2871, 3001, 3042, 3103, 3302, 3402 cm^{-1}.

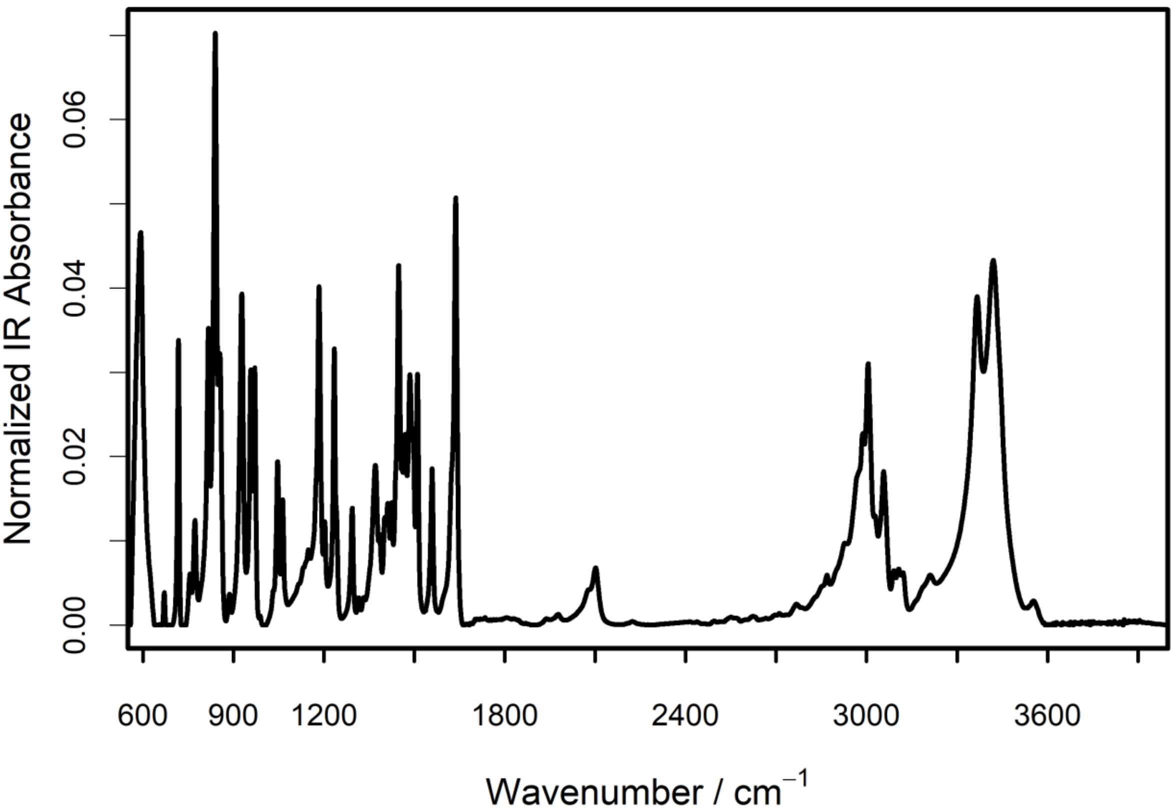

Figure S25. Baseline corrected and normalized ATR IR spectrum of BTMAPV crystals. Band positions: 593, 671, 718, 773, 817, 840, 855, 928, 958, 971, 1047, 1064, 1184, 1203, 1235, 1294, 1371, 1425, 1448, 1469, 1486, 1511, 1559, 1638, 2102, 3006, 3057, 3367, 3421 cm^{-1}.

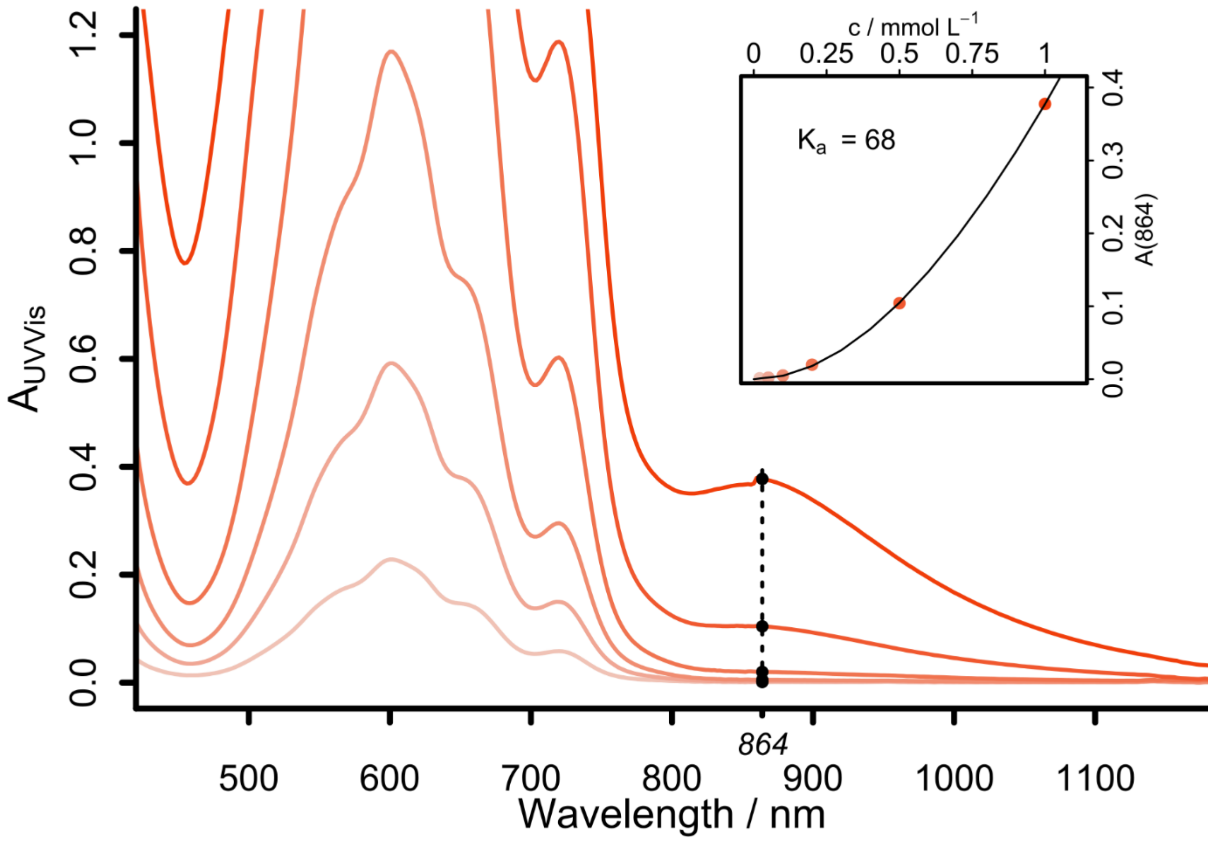

Figure S26. UV/Vis spectra of BTMAPVRC solutions of different concentrations. The band at 864 nm is associated with the formation of viologen radical cation dimers and can be used to determine the association constant between the monomer and its dimer using a non-linear fit (see inset).

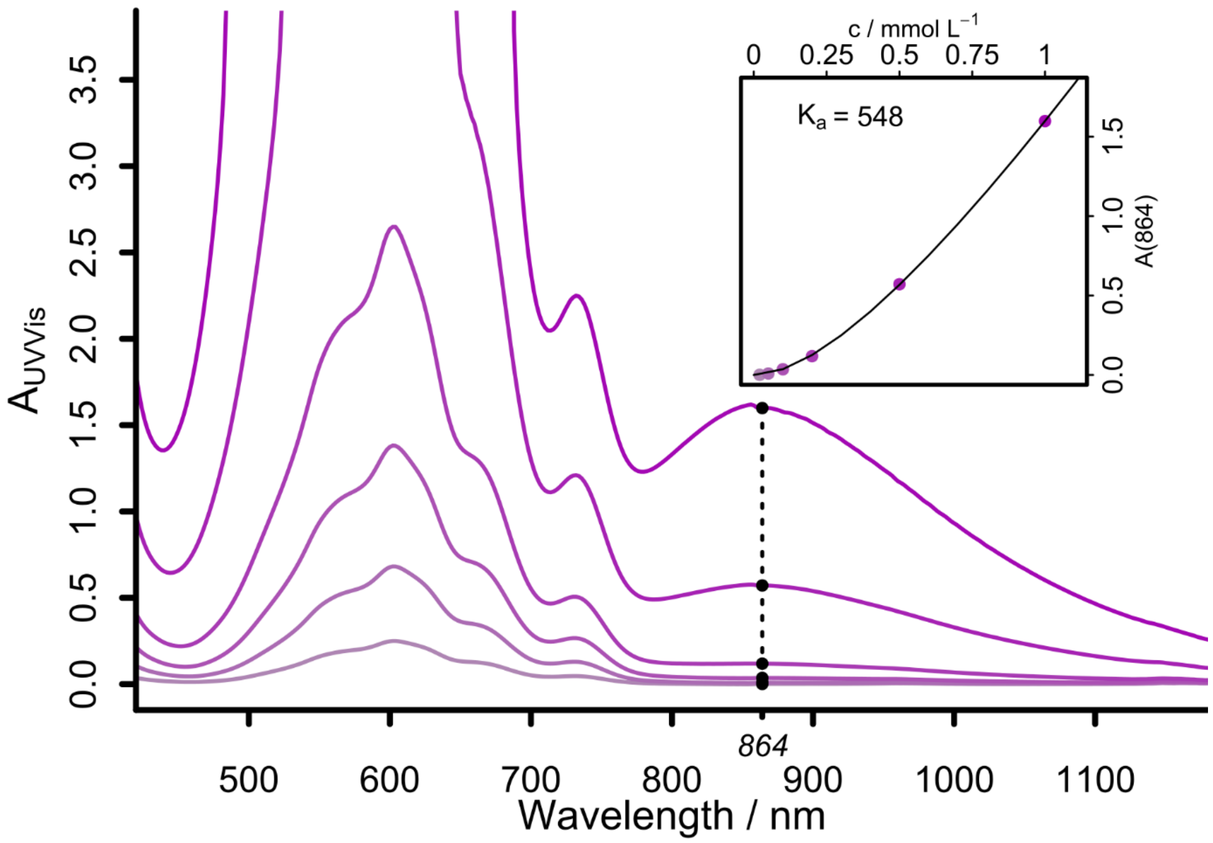

Figure S27. UV/Vis spectra of MVRC solutions of different concentrations. The band at 864 nm is associated with the formation of viologen radical cation dimers and can be used to determine the association constant between the monomer and its dimer using a non-linear fit (see inset).

8. References

(1) Janoschka, T.; Martin, N.; Hager, M. D.; Schubert, U. S. An Aqueous Redox-Flow Battery with High Capacity and Power: The TEMPTMA/MV System. *Angew. Chem. Int. Ed.* **2016**, *55*, 14427–14430.

(2) Liu, Y.; Goulet, M.-A.; Tong, L.; Liu, Y.; Ji, Y.; Wu, L.; Gordon, R. G.; Aziz, M. J.; Yang, Z.; Xu, T. A Long-Lifetime All-Organic Aqueous Flow Battery Utilizing TMAP-TEMPO Radical. *Chem* **2019**, *5*, 1861–1870.

(3) DeBruler, C.; Hu, B.; Moss, J.; Liu, X.; Luo, J.; Sun, Y.; Liu, T. L. Designer Two-Electron Storage Viologen Anolyte Materials for Neutral Aqueous Organic Redox Flow Batteries. *Chem* **2017**, *3*, 961–978.

(4) R Core team. *R: A Language and Environment for Statistical Computing*; R Foundation for Statistical Computing: Vienna, Austria, **2020**.

(5) Nolte, O.; Geitner, R.; Hager, M. D.; Schubert, U. S. IR Spectroscopy as a Method for Online Electrolyte State Assessment in RFBs. *Adv. Energy Mater.* **2021**, *11*, 2100931.

(6) Wickham, H. *ggplot2: Elegant Graphics for data Analysis*; Springer-Verlag: New York, **2016**.

(7) Wickham, H.; François, R.; Henry, L.; Müller, K. *dplyr: A Grammar of Data Manipulation*, **2021**.

(8) Auguie, B. *gridExtra: Miscellaneous Functions for "Grid" Graphics*, **2017**.

(9) Bruun, S. W.; Kohler, A.; Adt, I.; Sockalingum, G. D.; Manfait, M.; Martens, H. Correcting attenuated total reflection-Fourier transform infrared spectra for water vapor and carbon dioxide. *Appl. Spectrosc.* **2006**, *60*, 1029–1039.

(10) Ryan, C. G.; Clayton, E.; Griffin, W.; Sie, S.; Cousens, D. SNIP, a Statistics-Sensitive Background Treatment for the Quantitative Analysis of PIXE Spectra in Geoscience Applications. *Nucl. Instrum. Methods Phys. Res., Sect. B* **1988**, *34*, 396–402.

(11) Morhác, M. *Peaks: Peaks*, **2012**.

(12) Mullen, K. M. *ALS: Multivariate Curve Resolution Alternating Least Squares*, **2015**.

(13) Goulet, M.-A.; Aziz, M. J. Flow Battery Molecular Reactant Stability Determined by Symmetric Cell Cycling Methods. *J. Electrochem. Soc.* **2018**, *165*, A1466-A1477.

(14) Milshtein, J. D.; Barton, J. L.; Darling, R. M.; Brushett, F. R. 4-acetamido-2,2,6,6-tetramethylpiperidine-1-oxyl as a model organic redox active compound for nonaqueous flow batteries. *J. Power Sources* **2016**, *327*, 151–159.

(15) Mullen, K. M.; Ivo H. M. van Stokkum. *nnls: The Lawson-Hanson algorithm for non-negative least squares (NNLS)*, **2012**.

(16) Geraskina, M. R.; Dutton, A. S.; Juetten, M. J.; Wood, S. A.; Winter, A. H. The Viologen Cation Radical Pimer: A Case of Dispersion-Driven Bonding. *Angew. Chem. Int. Ed.* **2017**, *56*, 9435–9439.

(17) Tian, J.; Ding, Y.-D.; Zhou, T.-Y.; Zhang, K.-D.; Zhao, X.; Wang, H.; Zhang, D.-W.; Liu, Y.; Li, Z.-T. Self-assembly of three-dimensional supramolecular polymers through cooperative tetrathiafulvalene radical cation dimerization. *Chem. Eur. J.* **2014**, *20*, 575–584.

(18) Söntjens, S. H. M.; Sijbesma, R. P.; van Genderen, M. H. P.; Meijer, E. W. Stability and Lifetime of Quadruply Hydrogen Bonded 2-Ureido-4[1 H]-pyrimidinone Dimers. *J. Am. Chem. Soc.* **2000**, *122*, 7487–7493.
